# Heterointerface Engineering of Hierarchically Assembling Layered Double Hydroxides on Cobalt Selenide as Efficient Trifunctional Electrocatalysts for Water Splitting and Zinc‐Air Battery

**DOI:** 10.1002/advs.202104522

**Published:** 2022-01-12

**Authors:** Junnan Song, Ying Chen, Hongjiao Huang, Jiajun Wang, Shao‐Chu Huang, Yen‐Fa Liao, Amani E. Fetohi, Feng Hu, Han‐yi Chen, Linlin Li, Xiaopeng Han, K. M. El‐Khatib, Shengjie Peng

**Affiliations:** ^1^ College of Materials Science and Technology Nanjing University of Aeronautics and Astronautics Nanjing 210016 China; ^2^ Tianjin Key Laboratory of Composite and Functional Materials Key Laboratory of Advanced Ceramics and Machining Technology (Ministry of Education) School of Material Science and Engineering Tianjin University Tianjin 300072 China; ^3^ Joint School of National University of Singapore and Tianjin University International Campus of Tianjin University Binhai New City Fuzhou 350207 China; ^4^ Department of Materials Science and Engineering National Tsing Hua University Hsinchu 30013 Taiwan; ^5^ National Synchrotron Radiation Research Center Hsinchu 30013 Taiwan; ^6^ Chemical Engineering and Pilot Plant Department Engineering Research Institute National Research Centre 33 El‐Buhouth St. Dokki Cairo 12622 Egypt

**Keywords:** cobalt selenide, electrocatalysis, heterostructure, layered double hydroxide, Zn‐air battery

## Abstract

Engineering of structure and composition is essential but still challenging for electrocatalytic activity modulation. Herein, hybrid nanostructured arrays (HNA) with branched and aligned structures constructed by cobalt selenide (CoSe_2_) nanotube arrays vertically oriented on carbon cloth with CoNi layered double hydroxide (CoSe_2_@CoNi LDH HNA) are synthesized by a hydrothermal‐selenization‐hybridization strategy. The branched and hollow structure, as well as the heterointerface between CoSe_2_ and CoNi LDH guarantee structural stability and sufficient exposure of the surface active sites. More importantly, the strong interaction at the interface can effectively modulate the electronic structure of hybrids through the charge transfer and then improves the reaction kinetics. The resulting branched CoSe_2_@CoNi LDH HNA as trifunctional catalyst exhibits enhanced electrocatalytic performance toward oxygen evolution/reduction and hydrogen evolution reaction. Consequently, the branched CoSe_2_@CoNi LDH HNA exhibits low overpotential of 1.58 V at 10 mA cm^−2^ for water splitting and superior cycling stability (70 h) for rechargeable flexible Zn‐air battery. Theoretical calculations reveal that the construction of heterostructure can effectively lower the reaction barrier as well as improve electrical conductivity, consequently favoring the enhanced electrochemical performance. This work concerning engineering heterostructure and topography‐performance relationship can provide new guidance for the development of multifunctional electrocatalysts.

## Graphical abstract

Hierarchical CoSe_2_@CoNi‐layered double hydroxide (LDH) hybrid nanostructured arrays grown on carbon cloth (CC) are developed as trifunctional catalysts toward oxygen evolution/oxygen reduction/hydrogen evolution reaction. The coupling effect between CoSe_2_ nanotube and CoNi‐LDH nanosheets as well as the branched nanostructure enable CoSe_2_@CoNi‐LDH/CC hybrid nanostructured arrays with faster dynamics and more active sites, leading to satisfactory performance toward water splitting and Zn‐air batteries.

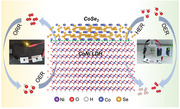

## 1 Introduction

Water splitting and metal‐air batteries have been considered as two promising energy technologies to alleviate energy dilemma.^[^ ^1^, ^2^, ^3^ ^]^ Generally, hydrogen evolution reaction (HER), oxygen evolution reaction (OER), as well as oxygen reduction reaction (ORR) are three crucial reactions in above energy technologies. Nevertheless, the sluggish kinetics in HER/OER/ORR due to the high overpotential in actual work greatly restricts the efficiency of operations.^[^ ^4^, ^5^ ^]^ Noble‐metal materials are currently the benchmark catalysts that can effectively reduce the catalytic overpotentials, however, high costs, poor stability, and the singularity of catalysis (Pt for ORR and HER, Ir/Ru for OER) hinder their widespread application.^[^ ^6^, ^7^, ^8^ ^]^ Therefore, low‐cost, and stable catalysts that can reversibly catalyze oxygen and hydrogen electrocatalysis are indispensable to improve the efficiency for water splitting and rechargeable metal‐air batteries.

Transition metal chalcogenides especially selenides exhibit high electrocatalytic performance owing to superior electrical conductivity, abundant 3d electrons configuration, and multiple morphologies, thereby being widely investigated.^[^ ^9^, ^10^, ^11^, ^12^ ^]^ Especially, the self‐supporting catalysts constructed by growing selenides on the conductive substrates such as carbon clothes (CC), on the one hand, could accelerate the diffusion of electrolyte and improve conductivity in the absence of binder;^[^ ^13^, ^14^ ^]^ on the other hand, could be directly used as air electrode for flexible electronic devices.^[^ ^15^, ^16^, ^17^ ^]^ These attractive characteristics play a key role in the improvement of electrochemical performance. Although cobalt selenide （CoSe） catalysts have shown potential energy applications, the electrocatalytic performance in terms of activity and cycle stability is still unsatisfactory. Therefore, it is still a great challenge to design CoSe‐based electrocatalysts that can simultaneously catalyze OER and ORR as well as HER since CoSe barely displays limited OER catalytic activity.

Recently, layered double hydroxide (LDH) materials are composed of versatile metal cation and charge‐balancing anions in the interlayer, showing potential applications for OER and ORR. Especially, heterostructure electrocatalysts based on LDH have shown superior electrocatalytic performance duo to improved physical or chemical properties by comparison with single component catalysts.^[^ ^18^, ^19^, ^20^, ^21^ ^]^ The heterojunction can contribute more exposed active sites and facilitate the reaction dynamics.^[^ ^22^, ^23^, ^24^ ^]^ Furthermore, the synergistic effect between single components enables the heterogeneous catalyst showing multifunctional characteristics, indicating that the heterostructure composite might be an ideal candidate as trifunctional catalyst toward HER/OER/ORR. In addition, engineering of structures is essential to further improve the catalytic properties by shortening transfer distance to enhance dynamics or introducing a large surface area to expose more active sites.^[^ ^25^, ^26^ ^]^ Therefore, it is expected that superior multifunctional electrocatalytic performance toward HER/OER/ORR can be achieved by rationally engineering heterostructured composites, which is consisted of metal selenides and metal LDH.

Herein, 3D strongly coupled ternary hybrid nanostructured arrays (HNA) in which branched and aligned CoSe_2_ nanotubes coated by CoNi LDH nanosheets, are successively grown on CC (CoSe_2_@CoNi LDH HNA) by a hydrothermal‐selenization‐hybridization strategy. In such a hybrid system, the hollow structure and strong coupling effect between branched CoSe_2_ nanotube arrays (B‐CoSe_2_) and CoNi LDH nanosheets enable CoSe_2_@CoNi LDH HNA with favorable mass transport and fast dynamics as well as abundant active sites for catalysis. Consequently, the branched CoSe_2_@CoNi LDH HNA (B‐CoSe_2_@CoNi LDH HNA) display highly efficient catalytic activity for OER (240 mV at 10 mA cm^−2^), HER (100 mV at 10 mA cm^−2^), and ORR (0.81 V for half‐wave potential). Furthermore, a two‐electrode electrolyzer with B‐CoSe_2_@CoNi LDH HNA as self‐supporting electrode only needs 1.58 V to attain the current density of 10 mA cm^−2^. Moreover, B‐CoSe_2_@CoNi LDH‐based Zn‐air battery shows a high‐power density of 181.5 mW cm^−2^, better than Pt/C catalyst (142.2 mW cm^−2^). The high performance of the hybrid catalyst demonstrates that reasonable structure design of electrocatalysts can lead to optimized surface and electronic configurations, resulting in superior multifunctional electrocatalytic activity.

## 2 Results and Discussion

**Figure 1:**
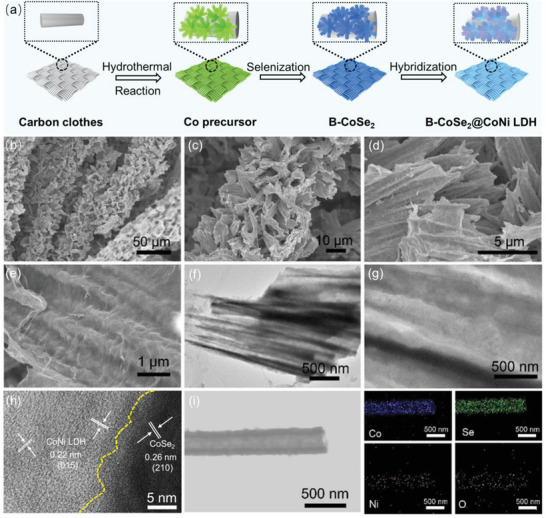
a) Schematic illustration of the synthetic process of B‐CoSe_2_@CoNi LDH HNA. and b,c) SEM images of the as‐synthesized B‐CoSe_2_@CoNi LDH HNA. d–f) High‐resolution SEM images of the B‐CoSe_2_@CoNi LDH HNA. g) TEM images of B‐CoSe_2_@CoNi LDH HNA. h) HRTEM image of B‐CoSe_2_@CoNi LDH HNA highlighting the interface between CoSe_2_ and CoNi LDH. i) Elemental mapping images of the B‐CoSe_2_@CoNi LDH HNA.

A delicate synthesis strategy of hydrothermal‐selenization‐hybridization is employed to fabricate the 3D hollow CoSe_2_@CoNi LDH HNA catalyst, which is specifically described in **Figure** **1**a. First, carbon cloth as a self‐supported substrate was employed to prepare Co precursors with branched morphologies by hydrothermal reaction (Figure S1, Supporting Information). Second, hollow CoSe_2_ nanoarrays with branched morphologies were obtained after selenization due to the diffusion process of Co ions driven by Kirkendall effect,^[^ ^27^, ^28^, ^29^ ^]^ which is briefly named as B‐CoSe_2_ (Figure S3, Supporting Information). It is worthwhile to mention that such hollow structure can provide abundant electrode/electrolyte contact interfaces as well as reduce the ion diffusion path for fast electrochemical kinetics. Moreover, the large void space in hollow structures facilitates the storage of a large amount of charge, which endows a high cycling ability. Finally, corresponding hierarchical B‐CoSe_2_@CoNi LDH HNA catalysts were obtained with the deposition of CoNi LDH on the surface of CoSe_2_ nanotubes. And Co precursors with vertical morphologies were obtained by controlling the amount of NH_4_F during hydrothermal reaction (Figure S2, Supporting Information). The corresponding samples after selenization and hybridization were named as V‐CoSe_2_ and V‐CoSe_2_@CoNi LDH HNA, respectively. The morphology and microstructure information are shown in Figure S4, Supporting Information. For comparison, CoSe_2_ and CoNi LDH were also prepared, see Experimental Section, Supporting Information for detailed experimental procedures.

As described in Figure 1b,c, the obtained B‐CoSe_2_@CoNi LDH HNA display 3D hierarchical structure, which are uniformly coated on the surface of CC in a large scale. It can be observed that CoSe_2_ presents branched 1D structure inherited from the Co precursor. The high‐magnification scanning electron microscope (SEM) images show that CoNi LDH nanosheets are fully grown on the surface of CoSe_2_ branched nanoarrays as backbone (Figure 1d,e). Transmission electron microscope (TEM) images in Figure 1f,g indicate the hollow structure of CoSe_2_ nanoarrays in B‐CoSe_2_@CoNi LDH HNA. The high‐resolution TEM image in Figure 1h shows an obvious interface between CoSe_2_ nanotubes and CoNi LDH, with the lattice spacings of 0.26 and 0.22 nm corresponding to the (210) and (015) planes of CoSe_2_ and CoNi LDH, respectively. Furthermore, Co, Ni, Se, and O elements are evenly distributed in B‐CoSe_2_@CoNi LDH HNA from the TEM element mapping (Figure 1i). V‐CoSe_2_@CoNi LDH HNA is composed of CoSe_2_ aligned nanoarrays coated by CoNi LDH nanosheets (Figure S6, Supporting Information). The independent CoNi LDH sample as reference shows the morphology of agglomerated microspheres (Figure S7, Supporting Information), indicating the strong chemical coupling between CoNi LDH and CoSe_2_ can promote the CoNi LDH nanosheets to dispersedly grow on the surface of CoSe_2_ nanotube arrays. The above results indicate that two different morphologies of CoSe_2_@CoNi LDH HNA catalysts with hollow structure and heterogeneous interface have been prepared successfully. The Brunauer–Emmett–Teller (BET) surface areas of as‐prepared B‐CoSe_2_, V‐CoSe_2_, B‐CoSe_2_@CoNi LDH HNA, V‐CoSe_2_@CoNi LDH HNA, and CoNi LDH are measured to be 6.47, 0.98, 26.38, 20.60, and 15.38 m^2^ g^−1^, respectively (Figure S7, Supporting Information). Compared with both CoSe_2_ and CoNi LDH, heterogeneous CoSe_2_@CoNi LDH HNA catalysts with larger BET surface area can expose more active sites. Especially, the B‐CoSe_2_@CoNi LDH HNA possesses largest surface area value, indicating the branched structure further increases the exposure of catalytically active sites.

**Figure 2:**
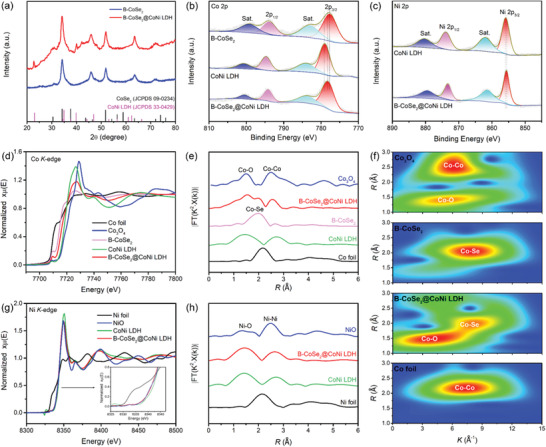
a) XRD spectra and b) XPS patterns of the Co 2p for B‐CoSe_2_, CoNi LDH, and B‐CoSe_2_@CoNi LDH HNA. c) XPS patterns of Ni 2p for CoNi LDH and B‐CoSe_2_@CoNi LDH HNA. d) The Co K‐edge XANES and e) Co K‐edge Fourier transform EXANES spectra of Co foil, Co_3_O_4_,B‐CoSe_2_, and B‐CoSe_2_@CoNi LDH HNA. f) The wavelet transforms of Co foil, B‐CoSe_2,_ B‐CoSe_2_@CoNi LDH HNA, and standard Co_3_O_4_. g) The Ni K‐edge XANES and h) Ni K‐edge Fourier transform EXANES spectra of Ni foil, NiO, CoNi LDH, and B‐CoSe_2_@CoNi LDH HNA.

The X‐ray diffraction (XRD) characterization was employed to identify the phase of B‐CoSe_2_@CoNi LDH HNA. As displayed in **Figure** **2**a, the characteristic peaks in the XRD pattern of B‐CoSe_2_@CoNi LDH HNA are assigned to CoSe_2_ (JCPDS 09‐0234), indicating that Co precursor was completely transformed into CoSe_2_ after selenization treatment. Besides the characteristic peaks of CoSe_2_, the other peaks can be assigned to the planes of CoNi LDH (JCPDS 33‐0429).^[^ ^30^, ^31^ ^]^ Simultaneously, the same characteristic peaks are observed in the XRD pattern of V‐CoSe_2_@CoNi LDH HNA (Figure S8, Supporting Information). The results demonstrate that CoSe_2_ and CoNi LDH are successfully integrated. The surface composition of B‐CoSe_2_@CoNi LDH HNA was then investigated via X‐ray photoelectron spectrometer (XPS) and X‐ray absorption fine structure (XAFS) characterization. The Co 2p spectrum of B‐CoSe_2_ deconvolutes with two peaks at 778.4 and 793.3 eV along with two satellite peaks at 783.1 and 799.3 eV (Figure 2b), corresponding to Co 2p_3/2_ and Co 2p_1/2_ of Co–Se bond,^[^ ^32^, ^33^ ^]^ while the peaks at 779.1 and 794.3 eV of Co 2p_3/2_ and Co 2p_1/2_ belong to Co–O bond for CoNi LDH. Obviously, the binding energies of Co 2p_3/2_ and Co 2p_1/2_ for B‐CoSe_2_@CoNi LDH are higher than that of B‐CoSe_2_, but lower than that of CoNi LDH. In the Ni region (Figure 2c), the peaks at 855.8 and 873.2 eV are assigned to Ni 2p_3/2_ and Ni 2p_1/2_ of Ni–O bond, along with two shakeup satellite peaks (861.9 and 880.7 eV).^[^ ^34^ ^]^ Obviously, a negative shift is observed for Ni 2p peaks of B‐CoSe_2_@CoNi LDH compared to that of CoNi LDH. As for Se 3d spectra (Figure S9, Supporting Information), the peaks at 54.6 and 55.4 eV of Se 3d_5/2_ and Se 3d_3/2_^[^ ^35^, ^36^ ^]^ for B‐CoSe_2_@CoNi LDH exhibit negative shift in comparison with that of B‐CoSe_2_. The O1s spectra present three peaks around 533, 531, and 529 eV, which are assigned to adsorbed H_2_O, O–H, and M–O bond, respectively. The bonds shift of Co, Ni, and Se are related to the formation of the heterojunction, confirming the existence of strong electronic interactions between B‐CoSe_2_ and CoNi LDH. These strong electronic interactions were induced by the redistribution of interface charge,^[^ ^37^, ^38^, ^39^ ^]^ which can result in faster charge/ion transfer for electrochemical reactions and then enhance the electrocatalytic performance.

XAFS characterization was subsequently employed to further investigate electronic interactions between B‐CoSe_2_ and CoNi LDH. The Co K‐edge X‐ray absorption near edge structure (XANES) spectra show that the adsorption edge of B‐CoSe_2_@CoNi LDH shifts to higher energy relative to B‐CoSe_2,_ but lower energy relative to CoNi LDH (Figure 2d), indicating the strong electronic interactions between B‐CoSe_2_ and CoNi LDH. Extended XAFS (EXAFS) of Co K‐edge was conducted to reveal the local chemical environment of Co sites. As described in Figure 2e, the spectrum of B‐CoSe_2_ shows a peak at 2.10 Å, assigned to Co–Se bond, while the spectrum of CoNi LDH displays a peak at 1.6 Å, corresponding to Co–O bond, in coincidence with XPS results. As for the spectrum of B‐CoSe_2_@CoNi LDH, both of Co–O and Co–Se bond can be detected. Furthermore, the Co–Se bond intensity of B‐CoSe_2_@CoNi LDH decreases in comparison to that of B‐CoSe_2_ nanotubes, indicating that the structural defects were induced by the LDH incorporation.^[^ ^40^, ^41^ ^]^ More intuitively, the wavelet transform (WT) analysis further demonstrate the coexistence of Co–O and Co–Se bonds in the spectrum of B‐CoSe_2_@CoNi LDH HNA (Figure 2f). Similarly, the Ni XANES spectra display the absorption edge of B‐CoSe_2_@CoNi LDH HNA slightly shifts to lower energy compared with the pure CoNi LDH (Figure 2g), implying the slight reduction of Ni oxidation states in B‐CoSe_2_@CoNi LDH HNA. In the EXAFS of Ni K‐edge (Figure 2h), two peaks corresponding to the Ni–O and Ni–Ni bonds can be found in the spectra of both B‐CoSe_2_@CoNi LDH and CoNi LDH. Therefore, the XPS and XANES analysis synergistically verify the existence of chemical coupling effects in B‐CoSe_2_@CoNi LDH HNA, which can alter the local electronic configurations of both CoSe_2_ and CoNi LDH. These modulated electronic structures of B‐CoSe_2_@CoNi LDH HNA can optimize the adsorption/desorption process of reaction intermediate, and further resulting in improved electrocatalytic properties.^[^ ^42^, ^43^, ^44^ ^]^

**Figure 3:**
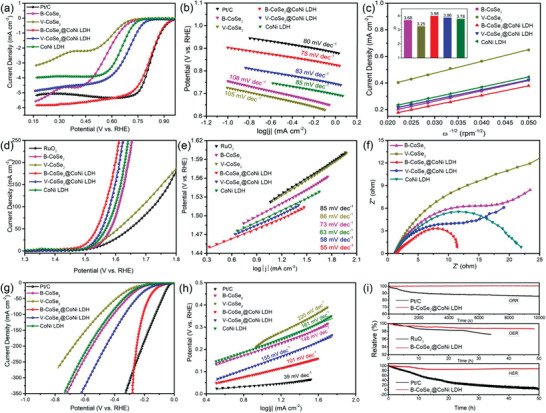
a–c) ORR, d–f) OER, and g,h) HER catalytic activity test of B‐CoSe_2_, V‐CoSe_2_, CoNi LDH, B‐CoSe_2_@CoNi LDH HNA, and V‐CoSe_2_@CoNi LDH HNA samples. ORR polarization curves (a), their corresponding Tafel data (b), and the *K–L* plots (c). The inset in (c) is their corresponding electron transferred numbers at 0.4 V versus RHE. OER polarization curves (d), their corresponding Tafel data (e), and electrochemical impedance spectroscopy (EIS) plots (f). HER polarization curves (g), their corresponding Tafel data (h). i) The stability test of B‐CoSe_2_@CoNi LDH HNA and Pt/C catalysts for ORR/OER/HER.

The multifunctional catalytic activity of as‐prepared catalysts as well as the relationship between morphology and properties were investigated. As displayed in **Figure** **3**a, B‐CoSe_2_@CoNi LDH HNA catalyst displays an ORR half‐potential of 0.81 V, which is close to commercial Pt/C catalyst (0.83 V), but much more positive than that of B‐CoSe_2_ (0.59 V), CoNi LDH (0.65 V), V‐CoSe_2_@CoNi LDH (0.69 V), and V‐CoSe_2_ (0.58 V). In addition, Tafel slope explains the intrinsic electrocatalytic performance of the catalyst under equilibrium environment, and ideal electrocatalysts are those having small Tafel slope.^[^ ^45^ ^]^ The B‐CoSe_2_@CoNi LDH HNA shows a smaller Tafel slope of 75 mV dec^−1^ in contrast to those of B‐CoSe_2_ (108 mV dec^−1^), V‐CoSe_2_ (105 mV dec^−1^), CoNi LDH (85 mV dec^−1^), and V‐CoSe_2_@CoNi LDH HNA (83 mV dec^−1^) (Figure 3b). The kinetics and electron transfer number (*n*) as two important evaluation parameters are further investigated. As shown in Figure S11, Supporting Information, the current density increases with the increase of the rotating speed due to the accelerated diffusion rates at high speed.^[^ ^46^, ^47^ ^]^ The corresponding *K–L* plots obtained from LSV curves present good linearity (Figure 3c), manifesting the first‐order reaction kinetics.^[^ ^48^, ^49^, ^50^ ^]^ And electron transfer number of B‐CoSe_2_@CoNi LDH HNA was calculated to be 3.9, indicating 4e^−^ transfer pathway for ORR (the inset in Figure 3c; Figure S12, Supporting Information). The OER catalytic activity of several samples is also explored and displays similar activity trends to ORR. As displayed in Figure 3d, B‐CoSe_2_@CoNi LDH HNA shows the potential of 1.48 V at 10 mA cm^−2^, which is lower than that of commercial RuO_2_ and most reported catalysts (Table S1, Supporting Information). The Tafel slope of B‐CoSe_2_@CoNi LDH HNA is 55 mV dec^−1^ (Figure 3e), which is close to that of V‐CoSe_2_@CoNi LDH HNA (58 mV dec^−1^), but much smaller than that of B‐CoSe_2_ (73 mV dec^−1^), V‐CoSe_2_ (86 mV dec^−1^), CoNi LDH (63 mV dec^−1^), and commercial RuO_2_ (85 mV dec^−1^), indicating the faster reaction kinetics on B‐CoSe_2_@CoNi LDH HNA. The Nyquist plots (Figure 3f) show a smaller semicircle of B‐CoSe_2_@CoNi LDH HNA than that of the other catalysts, indicating the lower electron transfer and mass transport resistances, corresponding to faster kinetics during OER process. The double‐layer capacitance (*C*_dl_) as a vital factor for the substantially enhanced OER activity is calculated to be 5.87, 1.92, 0.95, 2.96, and 4.37 mF cm^−2^ for B‐CoSe_2_@CoNi LDH HNA, B‐CoSe_2_, V‐CoSe_2_, CoNi LDH, and V‐CoSe_2_@CoNi LDH HNA, respectively (Figure S14, Supporting Information), revealing more abundant active sites of heterostructural B‐CoSe_2_@CoNi LDH HNA catalyst. Conclusively, B‐CoSe_2_@CoNi LDH HNA electrocatalyst shows comparable or (even) superior ORR/OER performance than commercial noble metal electrocatalysts in terms of reactivity and stability.

As a key parameter for water splitting, HER performance is further evaluated. B‐CoSe_2_@CoNi LDH HNA displays satisfactory HER electrocatalytic activity with the overpotential of 100 mV and the Tafel slope of 101 mV dec^−1^ (Figure 3g,h). This superior HER performance is also comparable among reported catalysts (Table S2, Supporting Information). In addition, B‐CoSe_2_@CoNi LDH HNA has high stability for all ORR/OER/HER, greatly outperforms commercial Pt/C and RuO_2_ catalysts (Figure 3i; Figure S15, Supporting Information). According to the above analysis, the OER/ORR/HER activities follow the order of B‐CoSe_2_@CoNi LDH > V‐CoSe_2_@CoNi LDH HNA > CoNi LDH >B‐CoSe_2_> V‐CoSe_2_. Compared to the single component catalyst, the heterostructure B‐CoSe_2_@CoNi LDH and V‐CoSe_2_@CoNi LDH HNA catalysts display remarkably enhanced catalytic performance. The enhanced performance is attributed to the strong chemical coupling between CoNi LDH and CoSe_2_, which can not only promote the CoNi LDH nanosheets to dispersedly grow on the CoSe_2_ nanotubes, but also regulate electronic structure on the surface of CoNi LDH and CoSe_2_ heterojunction. The derived heterostructure CoSe_2_@CoNi LDH catalysts with abundant active sites and accelerated interfacial reaction dynamics demonstrate superior performance for electrocatalysis. Especially, B‐CoSe_2_@CoNi LDH HNA exhibits better catalytic performance than V‐CoSe_2_@CoNi LDH HNA catalysts. As revealed by the BET analysis, the branched B‐CoSe_2_@CoNi LDH HNA possesses larger surface area than aligned V‐CoSe_2_@CoNi LDH HNA, which can expose more active sites for catalytic reaction. Noteworthy, the hollow structure also contributes to the improved performance by providing favorable mass transport via shortening the ion diffusion path. Furthermore, the large void space facilitates the storage of a large amount of charge, which endows a high cycling ability.

**Figure 4:**
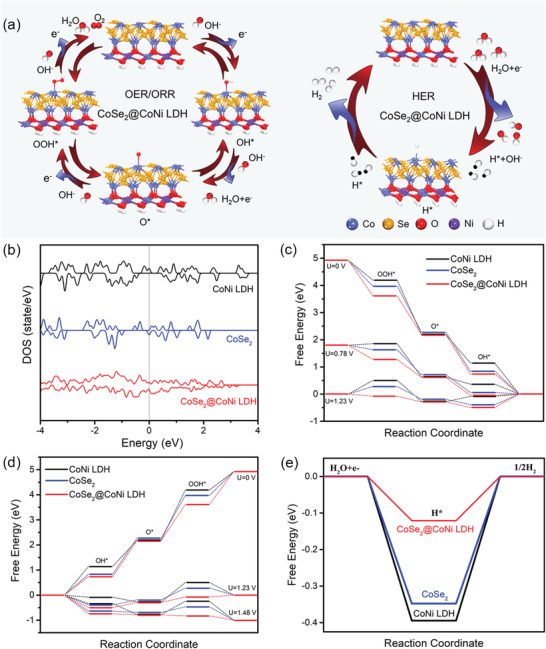
a) The models of adsorption configuration of ORR, OER, and HER on CoSe_2_@CoNi LDH HNA catalyst. b) The calculated PDOS of Co d‐orbital in three compounds. Free energy diagrams of CoSe_2_, CoNi LDH, and CoSe_2_@CoNi LDH HNA models for c) ORR, d) OER, and e) HER, respectively.

To elucidate the effect of heterogeneous interface on the activity of CoSe_2_@CoNi LDH HNA toward ORR/OER/HER, the DFT computation was performed. The specific calculation method is shown in Experimental Section, Supporting Information. The optimized atomic models of single CoSe_2_, CoNi LDH and CoSe_2_@CoNi LDH heterostructure are displayed in **Figure** **4**a and Figure S16, Supporting Information. ORR and OER are two inverse processes and involve four reaction steps, in which the intermediates (OOH*, O*, and OH* for ORR/OER, H* for HER) are adsorbed on Co sites. The projected density of states (PDOS) displays that the Co d‐orbital of CoSe_2_@CoNi LDH has a higher density state near the Fermi level than CoSe_2_ and CoNi LDH (Figure 4b). This indicates the enhanced electron transfer conferred by the construction of heterogeneous interface, in consistent with the EIS analysis (Figure 3f), contributing to the enhanced electrocatalytic properties. Moreover, the downhill free energy pathways in ORR at *U* = 0 V reveal that all electron transfer steps on CoSe_2_@CoNi LDH, CoSe_2_, and CoNi LDH can proceed spontaneously (Figure 4c). When the potential reaches 0.78 V (half‐wave potential of CoSe_2_@CoNi LDH HNA), the first step has become endothermic on CoNi LDH. The largest downhill free energy from the fifth reaction step manifests the rate‐determining step (RDS). With the potential increased to 1.23 V, the first step steps have been endothermic on both CoSe_2_ and CoNi LDH. By contrast, the step on CoSe_2_@CoNi LDH is still exothermic. Regarding OER (Figure 4d), the limiting barrier at RDS (O* to OOH*) is greatly reduced from the 1.70 eV for CoSe_2_ or 2.01 eV for CoNi LDH to the final 1.44 eV for CoSe_2_@CoNi LDH, accounting for the enhanced OER activity. For HER, the adsorption free energy of H* on CoSe_2_@CoNi LDH is −0.12 eV, which is lower than that on CoNi LDH (−0.39 eV) and CoSe_2_ (−0.35 eV) (Figure 4e), indicating smaller reaction barriers between adsorption and desorption processes achieving higher HER performance. The little free energy values demonstrated the hybridization of CoSe_2_ and CoNi LDH was conducive to alkaline ORR/OER/HER, which was consistent with the experimental results. The above DFT calculations display that heterostructure can effectively lower the reaction barrier as well as improve electrical conductivity, consequently favoring the enhanced electrochemical performance.

**Figure 5:**
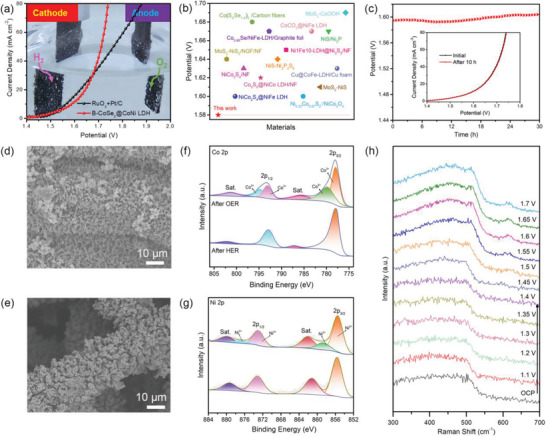
a) LSV curves of water splitting and photo of electrolyzer. b) Comparison of the water splitting performance with reported results. c) Stability tests of B‐CoSe_2_@CoNi LDH HNA based water electrolyzer device. d,e) SEM images; XPS spectra of f) Co 2p, and g) Ni 2p after water splitting processes for B‐CoSe_2_@CoNi LDH HNA. h) In situ Raman spectra of B‐CoSe2@CoNi LDH HNA at various potentials for OER process.

In view of the high OER and HER performance (Figure S17, Supporting Information), B‐CoSe_2_@CoNi LDH HNA was used as free‐standing electrode to assemble an electrolyzer for water splitting. As described in **Figure** **5**a, B‐CoSe_2_@CoNi LDH HNA (1.58 V at 10 mA cm^−2^) delivers comparable performance with Pt/C‐RuO_2_ (1.57 V at 10 mA cm^−2^). Moreover, B‐CoSe_2_@CoNi LDH HNA is also comparable to other heterostructure catalysts reported in the literature (Figure 5b; Table S3, Supporting Information). Durability as another important parameter in the practical application of electrolyzed water has also been evaluated through the chronovoltage response and cycle test. The LSV polarization curve of B‐CoSe_2_@CoNi LDH HNA remains almost unchanged after 10 h cycle test, indicating high stability. This is also confirmed by the timing‐voltage test in Figure 5c, in which the potential at 10 mA cm^−2^ shows no degradation in 30 h continuous working. By contrast, Pt/C‐RuO_2_ catalyst shows poor stability with a sharp activity drop within 8 h (Figure S18, Supporting Information).

The morphology, structure, and composition after electrochemical durability tests of the B‐CoSe_2_@CoNi LDH HNA sheet heterostructure were further explored. The branched morphologies of B‐CoSe_2_@CoNi LDH HNA are well maintained after water splitting (Figure 5d,e). Moreover, there is also no detectable change in the XRD pattern of the material after water splitting (Figure S19, Supporting Information). Furthermore, XPS analysis was carried out to analyze the composition of the B‐CoSe_2_@CoNi LDH HNA. As shown in high‐resolution spectrum of Co 2p, two new peaks derived from the Co^3+^ appear at the binding energy of 780 and 795 eV after OER process (Figure 5f), which could further confirm the formation of CoOOH. Similarly, the Ni 2p peaks (Figure 5g) also exhibit the same variation trend, indicating the possible structural evolution from Ni(OH)_2_ to NiOOH. In the case of Se 3d and O1s spectra (Figure S20, Supporting Information), the Se 3d signal disappears and the intensity of O–H increases, further demonstrating the possible formation of oxyhydroxide during water oxidation process. Similarly, the peaks appear at about 473, 508, and 615 cm^−1^ in in‐situ Raman spectra (Figure 5h) are assigned to the bending and stretching vibration mode of M–O in MOOH (M = Co, Ni), evidencing the oxidation of M(OH)_2_ to MOOH, which is consistent with the XPS results. In the process of HER, B‐CoSe_2_@CoNi LDH HNA nearly has no obvious changes compared to pristine B‐CoSe_2_@CoNi LDH HNA in terms of SEM, XRD, and XPS analysis, indicating that the structure is stable in the process of HER.

**Figure 6:**
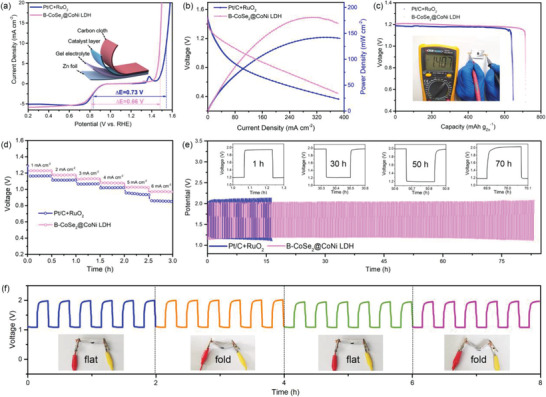
a) Bifunctional activities for B‐CoSe_2_@CoNi LDH HNA and Pt/C + RuO_2_ catalysts in 0.1 m KOH solution at 1600 rpm. b) Power density curves of CoSe_2_@CoNi LDH‐1 HNA and Pt/C + RuO_2_ catalysts for Zn‐air batteries. c) Specific capacities and d) discharging curves at various current densities of B‐CoSe_2_@CoNi LDH HNA and Pt/C + RuO_2_ catalysts. e) Cycling performance of flexible Zn‐air batteries based on B‐CoSe_2_@CoNi LDH HNA and Pt/C + RuO_2_ catalysts at 1 mA cm^−2^. f) Cycling stability test of the battery at different bending angles.

B‐CoSe_2_@CoNi LDH HNA shows only a difference of 0.66 V between the ORR E_1/2_ and the OER *E_j_*_= 10_, which is even smaller than that of Pt/C‐RuO_2_ (0.73 V) and other catalysts (Figure S21, Supporting Information). In view of the superior ORR and OER performance, a flexible Zn‐air battery was constructed using the B‐CoSe_2_@CoNi LDH HNA as a free‐standing aircathode (**Figure** **6**a). B‐CoSe_2_@CoNi LDH HNA‐based battery reveals higher power density and specific capacity (181.5 mW cm^−2^, 716.9 mAh g_Zn_^−1^) than those of Pt/C/RuO_2_ (Figure 6b,c), indicating the optimized performance of B‐CoSe_2_@CoNi LDH‐based Zn‐air devices. As displayed in the inset in Figure 6c, the battery based on B‐CoSe_2_@CoNi LDH HNA delivers an open‐circuit voltage of 1.407 V. Furthermore, the B‐CoSe_2_@CoNi LDH HNA‐based battery demonstrates higher discharge voltages than the Pt/C‐RuO_2_ at different current densities (Figure 6d). The superior rate performance of B‐CoSe_2_@CoNi LDH HNA cathode indicates enhanced ORR dynamics. Cycling stability is also evaluated by discharge–charge cycling test in Figure 6e, where the voltage gap of B‐CoSe_2_@CoNi LDH HNA‐based battery barely varies over 210 cycles (70 h) discharge–charge test, while the Pt/C‐RuO_2_‐based battery shows an obvious performance decay after 49 cycles. Such excellent stability is superior to precious metals and most reported Zn‐air battery catalysts (Table S4, Supporting Information). As a proof of concept, an electronic screen displaying NUAA (≈3 V) can be powered by three batteries connected in series, and the electronic screen can still stay on even if the batteries are bent at any angle (Figure S23, Supporting Information). This considerable flexibility is also reflected in Figure 6f, the discharge–charge plateaus maintain extremely stable at different bending angles.

## 3 Conclusions

In summary, a novel 3D hierarchical CoSe_2_@CoNi LDH HNA with branched and aligned CoSe_2_ nanotube arrays coated by CoNi LDH nanosheets grown on CC have been controllably prepared by a stepwise hydrothermal‐selenization‐hybridization strategy. The resulting 3D branched B‐CoSe_2_@CoNi LDH HNA with high specific surface area, hierarchical pores, and conductive heterostructure displays promising trifunctional catalytic performance. It is demonstrated that a two‐electrode electrolyzer with CoSe_2_@CoNi LDH‐1 HNA as self‐supporting electrode only needs 1.58 V to drive a current density of 10 mA cm^−2^. Furthermore, rechargeable Zn‐air battery with B‐CoSe_2_@CoNi LDH‐1 HNA as air electrode displays excellent reversibility and stability, which is superior to most reported rechargeable flexible Zn‐air batteries. Experimental results and theoretical calculations reveal that the superior electrochemical performance is attributed to the heterogeneous interface between the CoSe_2_ hollow nanotubes and CoNi LDH nanosheets, the favorable branched morphology as well as the 3D hollow structure, which endows B‐CoSe_2_@CoNi LDH HNA with accelerated reaction kinetics, abundant active sites, and reasonable adsorption energy toward the reactant. It is expected that this work can provide insight to rational design and structure engineering of efficient and multifunctional electrocatalysts for various energy storage and conversion technologies.

## Conflict of Interest

The authors declare no conflict of interest.

## Supporting information

Supporting Information

## Data Availability

The data that support the findings of this study are available from the corresponding author upon reasonable request.

## References

[1] X. Wang , T. Ouyang , L. Wang , J. Zhong , T. Ma , Z. Liu , Angew. Chem., Int. Ed. 2019, 131, 13425.

[2] D. C. Nguyen , D. T. Tran , T. L. L. Doan , D. H. Kim , N. H. Kim , J. H. Lee , Adv. Energy Mater. 2020, 10, 1903289.

[3] C. Lai , J. Fang , X. Liu , M. Gong , T. Zhao , T. Shen , K. Wang , K. Jiang , D. Wang , Appl. Catal., B 2021, 285, 119856.

[4] C. Liang , P. Zou , A. Nairan , Y. Zhang , J. Liu , K. Liu , S. Hu , F. Kang , H. Fan , C. Yang , Energy Environ. Sci. 2020, 13, 86.

[5] H. Yang , L. Gong , H. Wang , C. Dong , J. Wang , K. Qi , H. Liu , X. Guo , B. Xia , Nat. Commun. 2020, 11, 5075. 33033245 10.1038/s41467-020-18891-x PMC7545195

[6] J. Diao , Y. Qiu , S. Liu , W. Wang , K. Chen , H. Li , W. Yuan , Y. Qu , X. Guo , Adv. Mater. 2020, 32, 1905679. 10.1002/adma.201905679 31736168

[7] T. Ouyang , X.‐T. Wang , X.‐Q. Mai , A.‐N. Chen , Z.‐Y. Tang , Z.‐Q. Liu , Angew. Chem., Int. Ed. 2020, 59, 11948. 10.1002/anie.202004533 32337761

[8] H. Wang , Y. Cui , Carbon Energy 2019, 1, 13.

[9] J. Song , S. Qiu , F. Hu , Y. Ding , S. Han , L. Li , H.‐Y. Chen , X. Han , C. Sun , S. Peng , Adv. Funct. Mater. 2021, 31, 2100618.

[10] W. Zhong , Z. Wang , N. Gao , L. Huang , Z. Lin , Y. Liu , F. Meng , J. Deng , S. Jin , Q. Zhang , L. Gu , Angew. Chem., Int. Ed. 2020, 59, 22743. 10.1002/anie.202011378 32896011

[11] H. Sun , Z. Yan , F. Liu , W. Xu , F. Cheng , J. Chen , Adv. Mater. 2020, 32, 1806326. 10.1002/adma.201806326 30932263

[12] K. N. Dinh , Y. Sun , Z. Pei , Z. Yuan , A. Suwardi , Q. Huang , X. Liao , Z. Wang , Y. Chen , Q. Yan , Small 2020, 16, 1905885. 10.1002/smll.201905885 32243082

[13] Y. Yan , Y. Xu , B. Zhao , Y. Xu , Y. Gao , G. Chen , W. Wang , B. Xia , J. Mater. Chem. A 2020, 8, 5070.

[14] Z. Zhao , Z. Yuan , Z. Fang , J. Jian , J. Li , M. Yang , C. Mo , Y. Zhang , X. Hu , P. Li , S. Wang , W. Hong , Z. Zheng , G. Ouyang , X. Chen , D. Yu , Adv. Sci. 2018, 5, 1800760. 10.1002/advs.201800760 PMC6299824 30581696

[15] X. Han , W. Zhang , X. Ma , C. Zhong , N. Zhao , W. Hu , Y. Deng , Adv. Funct. Mater. 2019, 31, 1808281. 10.1002/adma.201808281 30873660

[16] Y. Zhang , H. Sun , Y. Qiu , X. Ji , T. Ma , F. Gao , Z. Ma , B. Zhang , P. Hu , Carbon 2019, 144, 370.

[17] S. Han , Y. Hao , Z. Guo , D. Yu , H. Huang , F. Hu , L. Li , H.‐Y. Chen , S. Peng , Chem. Eng. J. 2020, 401, 126088.

[18] L. Zhou , C. Zhang , Y. Zhang , Z. Li , M. Shao , Adv. Funct. Mater. 2021, 31, 2009743.

[19] L. Ren , C. Wang , W. Li , R. Dong , H. Sun , N. Liu , B. Geng , Electrochim. Acta 2019, 318, 42.

[20] T. Zhang , L. Hang , Y. Sun , D. Men , X. Li , L. Wen , X. Lyu , Y. Li , Nanoscale Horiz. 2019, 4, 1132.

[21] B. H. R. Suryanto , Y. Wang , R. K. Hocking , W. Adamson , C. Zhao , Nat. Commun. 2019, 10, 5599. 31811129 10.1038/s41467-019-13415-8 PMC6898202

[22] Q. Xu , H. Jiang , H. Zhang , Y. Hu , C. Li , Appl. Catal., B 2019, 242, 60.

[23] Y. Guo , P. Yuan , J. Zhang , H. Xia , F. Cheng , M. Zhou , J. Li , Y. Qiao , S. Mu , Q. Xu , Adv. Funct. Mater. 2018, 28, 1805641.

[24] G. Liu , M. Wang , Y. Wu , N. Lia , F. Zhao , Q. Zhao , J. Li , Appl. Catal., B 2020, 260, 118199.

[25] J. Zhou , L. Yu , Q. Zhu , C. Huang , Y. Yu , J. Mater. Chem. A 2019, 7, 18118.

[26] X. Yu , G. Chen , Y. Wang , J. Liu , K. Pei , Y. Zhao , W. You , L. Wang , J. Zhang , L. Xing , J. Ding , G. Ding , M. Wang , R. Che , Nano Res. 2020, 13, 437.

[27] Y. Wu , H. Wang , S. Ji , B. G. Pollet , X. Wang , R. Wang , Nano Res. 2020, 13, 2098.

[28] T. Zhang , F. Meng , Y. Cheng , N. Dewangan , G. W. Ho , S. Kawi , Appl. Catal., B 2021, 286, 119853.

[29] W. Wang , Y. Lu , M. Zhao , R. Luo , Y. Yang , T. Peng , H. Yan , X. Liu , Y. Luo , ACS Nano 2019, 13, 12206. 31536322 10.1021/acsnano.9b06910

[30] G. Xiong , Y. Chen , Z. Zhou , F. Liu , X. Liu , L. Yang , Q. Liu , Y. Sang , H. Liu , X. Zhang , J. Jia , W. Zhou , Adv. Funct. Mater. 2021, 31, 2009580.

[31] J. Dai , D. Zhao , W. Sun , X. Zhu , L. Ma , Z. Wu , C. Yang , Z. Cui , L. Li , S. Chen , ACS Catal. 2019, 9, 10761.

[32] C. Huang , D. Wu , P. Qin , K. Ding , C. Pi , Q. Ruan , H. Song , B. Gao , H. Chen , P. K. Chu , Nano Energy 2020, 73, 104788.

[33] L. Yang , H. Ren , Q. Liang , K. N. Dinh , R. Dangol , Q. Yan , Small 2020, 16, 1906766. 10.1002/smll.201906766 31985171

[34] P. Zhou , X. Lv , D. Xing , F. Ma , Y. Liu , Z. Wang , P. Wang , Z. Zheng , Y. Dai , B. Huang , Appl. Catal., B 2020, 263, 118330.

[35] W. He , L. Han , Q. Hao , X. Zheng , Y. Li , J. Zhang , C. Liu , H. Liu , H. L. Xin , ACS Energy Lett. 2019, 4, 2905.

[36] F. Li , G.‐F. Han , H.‐J. Noh , J.‐P. Jeon , I. Ahmad , S. Chen , C. Yang , Y. Bu , Z. Fu , Y. Lu , J.‐B. Baek , Nat. Commun. 2019, 10, 4060. 31492875 10.1038/s41467-019-12012-z PMC6731251

[37] X. Li , H. Wu , A. M. Elshahawy , L. Wang , S. J. Pennycook , C. Guan , J. Wang , Adv. Funct. Mater. 2018, 28, 1800036.

[38] C. Zhao , L. Tian , Z. Zou , Z. Chen , H. Tang , Q. Liu , Z. Lin , X. Yang , Appl. Catal., B 2020, 268, 118445.

[39] Q. Liu , Z. Xue , B. Jia , Q. Liu , K. Liu , Y. Lin , M. Liu , Y. Li , G. Li , Small 2020, 16, 2002482. 10.1002/smll.202002482 32627945

[40] K. Xiao , S.‐L. Zhao , M. Cao , L. Zhang , N. Li , Z.‐Q. Liu , J. Mater. Chem. A 2020, 8, 23257.

[41] Y. Wang , Y. Zhang , Z. Liu , C. Xie , S. Feng , D. Liu , M. Shao , S. Wang , Angew. Chem., Int. Ed. 2017, 129, 5961. 10.1002/anie.201701477 28429388

[42] T. Liu , A. Li , C. Wang , W. Zhou , S. Liu , L. Guo , Adv. Mater. 2018, 30, 1803590. 10.1002/adma.201803590 30285280

[43] X. Han , X. Ling , Y. Wang , T. Ma , C. Zhong , W. Hu , Y. Deng , Angew. Chem., Int. Ed. 2019, 58, 5359. 10.1002/anie.201901109 30790406

[44] Z. Liang , W. Zhou , S. Gao , R. Zhao , H. Zhang , Y. Tang , J. Cheng , T. Qiu , B. Zhu , C. Qu , W. Guo , Q. Wang , R. Zou , Small 2020, 16, 1905075. 10.1002/smll.201905075 31814261

[45] A. Ali , P. K. Shen , Carbon Energy 2020, 2, 99.

[46] Z. Sun , Y. Wang , L. Zhang , H. Wu , Y. Jin , Y. Li , Y. Shi , T. Zhu , H. Mao , J. Liu , C. Xiao , S. Ding , Adv. Funct. Mater. 2020, 30, 1910482.

[47] C.‐C. Hou , L. Zou , L. Sun , K. Zhang , Z. Liu , Y. Li , C. Li , R. Zou , J. Yu , Q. Xu , Angew. Chem., Int. Ed. 2020, 132, 7454. 10.1002/anie.202002665 32153103

[48] Y. Hu , Y. Lu , X. Zhao , T. Shen , T. Zhao , M. Gong , K. Chen , C. Lai , J. Zhang , H. L. Xin , D. Wang , Nano Res. 2020, 13, 2365.

[49] H. Hu , J. Wang , B. Cui , X. Zheng , J. Lin , Y. Deng , X. Han , Angew. Chem., Int. Ed. 2022, 61, e202114441. 10.1002/anie.202114441 34806271

[50] Z. Y. Wang , S. D. Jiang , C. Q. Duan , D. Wang , S. H. Luo , Y. G. Liu , Rare Met. 2020, 39, 1383.

