# Supplementary material for: Heterointerface Engineering of Hierarchically Assembling Layered Double Hydroxides on Cobalt Selenide as Efficient Trifunctional Electrocatalysts for Water Splitting and Zinc‐Air Battery
Source: Adv Sci (Weinh). 2022 Jan 12;9(6):2104522. doi: 10.1002/advs.202104522 (PMC8867188; doi:10.1002/advs.202104522)
Supplement: Supplementary file 1 — Supporting Information [file ADVS-9-2104522-s001.pdf]

ADVANCED SCIENCE
Open Access

Supporting Information

for *Adv. Sci.,* DOI: 10.1002/advs.202104522

Heterointerface Engineering of Hierarchically Assembling Layered Double Hydroxides on Cobalt Selenide as Efficient Trifunctional Electrocatalysts for Water Splitting and Zinc-Air Battery

Junnan Song, Ying Chen, Hongjiao Huang, Jiajun Wang, Shao-Chu Huang, Yen-Fa Liao, Amani E. Fetohi, Feng Hu, Han-yi Chen, Linlin Li, Xiaopeng Han,* K. M. El-Khatib, and Shengjie Peng**

Supporting Information

Heterointerface Engineering of Hierarchically Assembling Layered Double Hydroxides on Cobalt Selenide as Efficient Trifunctional Electrocatalysts for Water Splitting and Zinc-Air Battery

Junnan Song, Ying Chen, Hongjiao Huang, Jiajun Wang, Shao-Chu Huang, Yen-Fa Liao, Amani E. Fetohi, Feng Hu, Han-yi Chen, Linlin Li, Xiaopeng Han,* K. M. El-Khatib, and Shengjie Peng**

J. Song, Y. Chen, H. Huang, Prof. F. Hu, Prof. L. Li, Prof. S. Peng
College of Materials Science and Technology, Nanjing University of Aeronautics and Astronautics, Nanjing 210016, China.
E-mail: lilinlin@nuaa.edu.cn; pengshengjie@nuaa.edu.cn
Dr. J Wang, Prof. X. Han
Tianjin Key Laboratory of Composite and Functional Materials, Key Laboratory of Advanced Ceramics and Machining Technology (Ministry of Education), School of Material Science and Engineering, Tianjin University, Tianjin 300072, China
E-mail: xphan@tju.edu.cn
Dr. J Wang
Joint School of National University of Singapore and Tianjin University, International Campus of Tianjin University, Binhai New City, Fuzhou 350207, China
S.-C. Huang, Prof. H.-Y. Chen
Department of Materials Science and Engineering, National Tsing Hua University
Hsinchu 30013, Taiwan
Prof. Y-F. Liao
National Synchrotron Radiation Research Center, Hsinchu 30013, Taiwan.
A. E. Fetohi, K. M. El-Khatib
Chemical Engineering & Pilot Plant Department, Engineering Research Institute, National Research Centre, 33 El-Buhouth St., Dokki, Cairo, 12622, Egypt

Experimental Section

Raw Chemicals: Selenium (Se) powder, urea ($CO(NH_2)_2$), sodium borohydride ($NaBH_4$), cobalt chloride hexahydrate ($CoCl_2 \cdot 6H_2O$), cobalt nitrate hexahydrate (Co $(NO_3)_2 \cdot 6H_2O$), nickel nitrate hexahydrate (Ni $(NO_3)_2 \cdot 6H_2O$), cyclohexamethylenetetramine (HMT), absolute ethanol and ammonium fluoride (NH_4F) were purchased from Nanjing Chemical Reagent Co., Ltd and used without further purification.

Synthesis of Co precursors: Co precursors with branched and aligned structure were obtained under the control of NH_4F by hydrothermal methods [1, 2]. Specifically, $CoCl_2 \cdot 6H_2O$ (1.0 g), $CO(NH_2)_2$ (0.3 g) and NH_4F (0.3 g) were added into 30 mL of deionized water with stirring in 50 mL autoclave. Then the pre-treated CC were immersed in the mixed solution. The autoclave was heated at 120 °C for 10 h. When the reaction was complete, CC loaded with Co precursor was washed with deionized water and ethanol. Finally, Co precursor with branched structure was obtained after drying. By contrast, the Co precursor with aligned structure was prepared in the similar way only by varying the amount of NH_4F as 0.1 g.

Synthesis of $CoSe_2$: $CoSe_2$ nanotube arrays were obtained by selenization of Co precursor. Specifically, Se powder (0.30 g) and $NaBH_4$ (0.35 g) were dissolved into 30 mL of deionized water in a 50 mL reaction kettle. Subsequently, Co precursors were added and the reaction kettle was heated at 120 °C for 10 h. After cooling to room temperature, CC loaded with $CoSe_2$ nanotube arrays was washed with deionized water and ethanol. Finally, the branched $CoSe_2$ sample was obtained, denoted as B-$CoSe_2$. The $CoSe_2$ with the vertical structure was synthesized in the similar approach by changing the adding amount of NH_4F, denoted as V-$CoSe_2$.

Synthesis of $CoSe_2$@ CoNi LDH HNA: In detail, Co $(NO_3)_2 \cdot 6H_2O$ (0.03 g) and Ni $(NO_3)_2 \cdot 6H_2O$ (0.05 g) were dissolved into ethanol (15 mL) and water (15 mL) mixed solution

under stirring. Then HMT (0.1 g) was added and the mixed solution was poured into a 50 mL reaction kettle. Subsequently, B-$CoSe_2$ were added and the reaction kettle was heated at 80 °C for 10 h. After cooling to room temperature, moist CC were washed with deionized water and ethanol respectively, then dried at 60 °C. The synthesized $CoSe_2$@CoNi LDH HNA with branched structure was denoted as B-$CoSe_2$@CoNi LDH HNA. While V-$CoSe_2$ was added, the obtained $CoSe_2$@CoNi LDH with aligned structure was denoted as V-$CoSe_2$@CoNi LDH HNA. CoNi LDH sample as reference was prepared in the same way without adding $CoSe_2$.

Materials Characterizations: Morphology and microstructure of samples were observed via field-emission scanning electron microscope (FESEM, Regulus 8100) and transmission electron microscope (TEM, FEI Tecnai G2 F20) as well as high resolution TEM (HRTEM). TEM mapping was also conducted on FEI Tecnai G2 F20. The Brunauer–Emmett–Teller (BET) surface area were determined using nitrogen adsorption–desorption carried by the Surface Area (V-Sorb 2800P, Gold APP Instruments, China). X-ray diffraction (XRD) data acquired from Bruker D8 Advance instrument was used to explore the crystal structure. X-ray photoelectron spectrometer (XPS, Escalab 250Xi) with Al Kα X-rays as the excitation source were performed to investigate the surface composition of the samples. The extended X-ray absorption fine structure (EXAFS) was measured at Taiwan Photon Source (TPS44A1, TLS17C1) beam line, 44A Quick-scanning X-ray absorption spectroscopy (XAS), in National Synchrotron Radiation Research Center (NSRRC), Hsinchu, Taiwan. WT EXAFS software was applied to calculate the wavelet transformations. The in-suit Raman spectra were collected by RENISHAW equipped with an excitation wavelength of 633 nm.

Assembly of Solid-State Zn-Air Battery: Firstly, Acrylic acid (2.20 g) and N, N′-methylene-bisacrylamide (0.01 g) were dispersed by ultrasound to form a homogeneous solution. Then, 8.4 $moL.L^{-1}$ KOH solution was added dropwise and stirred continuously under ice bath. Subsequently, 0.3 M $K_2S_2O_8$ solution (80 μL) was added and stirred for 30 s. Finally,

the mixture was poured into a glass mold to obtain the thick gel thin film. The carbon cloth covered with $CoSe_2$@CoNi LDH-1 HNA catalysts (effective area of 1 cm^2) was directly used as air electrodes. The solid-state zinc-air battery was assembled via a layer-by-layer method. Zinc foil and carbon cloth covered with catalyst were placed on the two sides of the gel thin film. Finally, it was packed with aluminum film.

Calibration of RHE electrode: Ag/AgCl was used as reference electrode for all electrochemical measurements. However, all measured potentials in this work referred to the reversible hydrogen electrode (RHE). The calibration was performed in the high purity hydrogen saturated electrolyte with a Pt wire as the working electrode. CVs were run at a scan rate of 1 mV s−1, and the average of the two potentials at which the current crossed zero was taken to be the thermodynamic potential for the hydrogen electrode reactions.

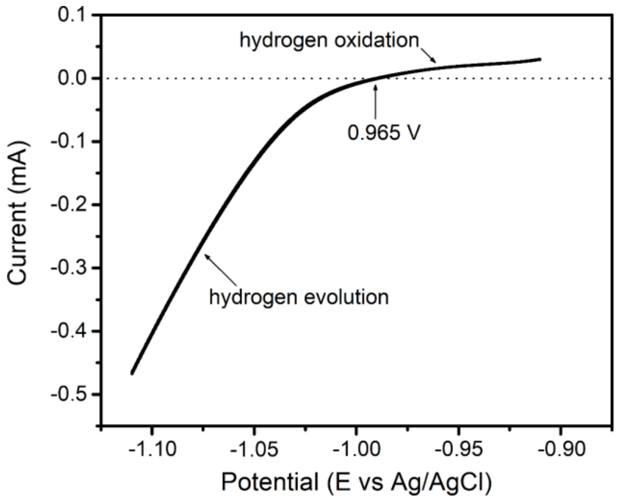

So, in 0.1 M KOH, E (RHE) = E (Ag/AgCl) + 0.965 V.

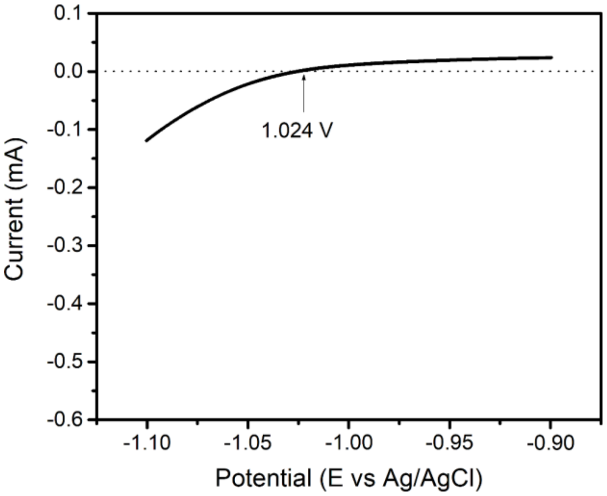

So, in 1 M KOH, E (RHE) = E (Ag/AgCl) + 1.024 V.

Electrochemical measurements: All the electrochemical tests were carried out with an Autolab working station. And all the potentials were referenced to RHE. The OER and HER tests were performed in 1.0 M KOH solution, with a catalyst decorated nickel foam or glassy carbon as working electrode, a platinum foil and an Ag/AgCl electrode as counter electrode and reference electrode, respectively. While the ORR test was performed in O_2-saturated 0.1 M KOH solution, with a glassy carbon electrode (diameter: 5 mm) coated with catalyst inks as working electrode, a carbon rod and an Ag/AgCl electrode as counter electrode and reference electrode, respectively. Polarization curves measurements of Zn-air batteries were also carried out on an Autolab electrochemical working station. Battery tests were carried out under atmospheric conditions with a Neware BTS3000n battery testing system.

Computational Calculations: We have the first-principles [3] were employed to perform all spin-polarization density functional theory (DFT) calculations within the generalized gradient approximation (GGA) using the Perdew-Burke-Ernzerhof (PBE) [4] formulation. We have chosen the projected augmented wave (PAW) potentials [5,6] to describe the ionic cores and take valence electrons into account using a plane wave basis set with a kinetic energy cutoff of 450 eV. Partial occupancies of the Kohn−Sham orbitals were allowed using the

Gaussian smearing method and a width of 0.05 eV. The electronic energy was considered self-consistent when the energy change was smaller than 10^{-4} eV. A geometry optimization was considered convergent when the energy change was smaller than 0.05 eV $Å^{-1}$. The vacuum spacing in a direction perpendicular to the plane of the structure is 18 Å. The Brillouin zone integration is performed using 2×2×1 Monkhorst-Pack k-point sampling for a structure. Finally, the adsorption energies (Eads) were calculated as $E_{ads}= E_{ad/sub} -E_{ad} -E_{sub}$, where $E_{ad/sub}$, E_{ad}, and E_{sub} are the total energies of the optimized adsorbate/substrate system, the adsorbate in the structure, and the clean substrate, respectively. The free energy was calculated using the equation:

$$G = E + ZPE - TS$$

where G, E, ZPE and TS are the free energy, total energy from DFT calculations, zero-point energy and entropic contributions, respectively. In our structure, the NiCo LDH (001) surface and $CoSe_2$ (211) surface had been established using first-principles from the bulk structure.

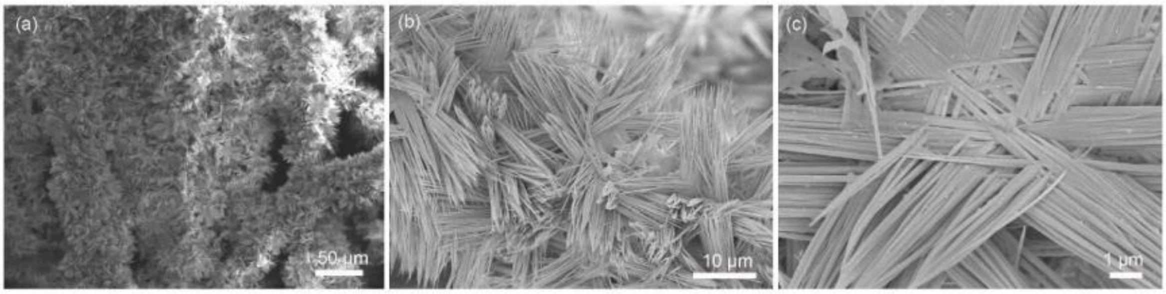

Figure S1. SEM of branched Co precursor.

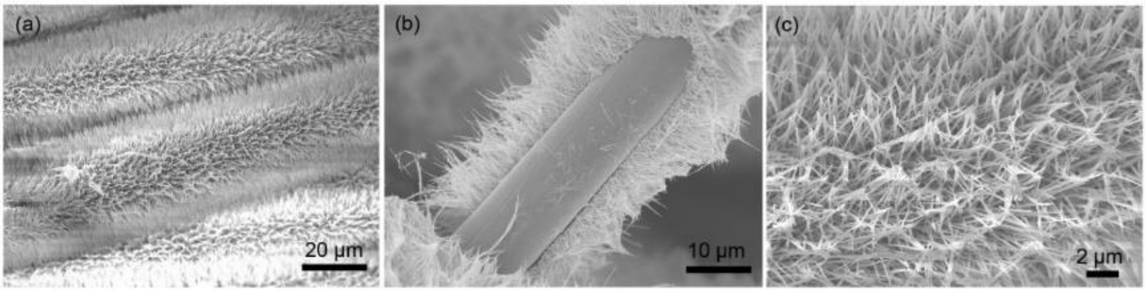

Figure S2. SEM of aligned Co precursor. Co precursors with two different morphologies were obtained through hydrothermal reaction. One is aligned nanoarray, and the other is branched array (Figure S1).

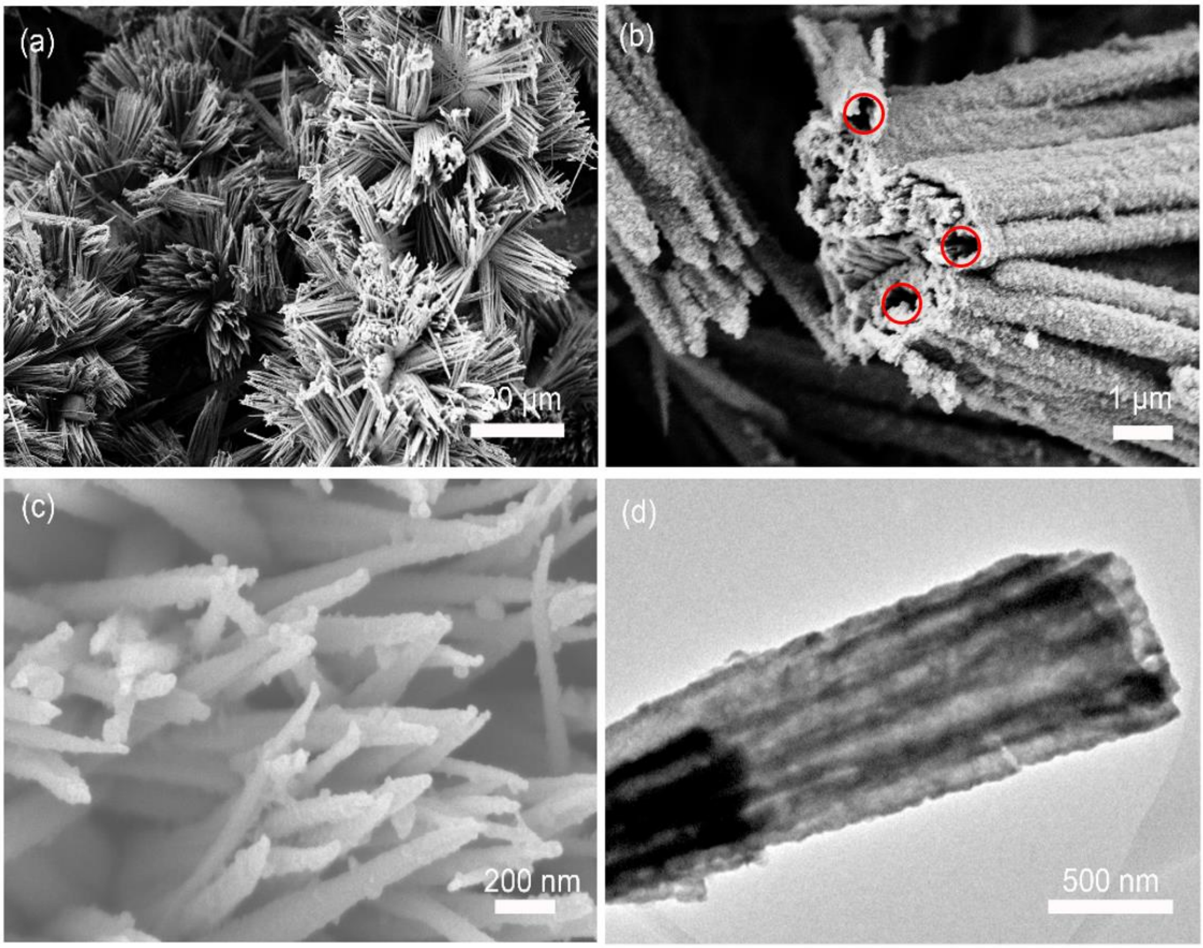

Figure S3. (a) and (b) Low-resolution SEM images of B-$CoSe_2$. (c) High resolution SEM images of B-$CoSe_2$. (d) TEM images of B-$CoSe_2$. After selenization process, hollow nanotube arrays were obtained, which is due to the diffusion process of Co ions during the selenization driven by Kirkendall effect.

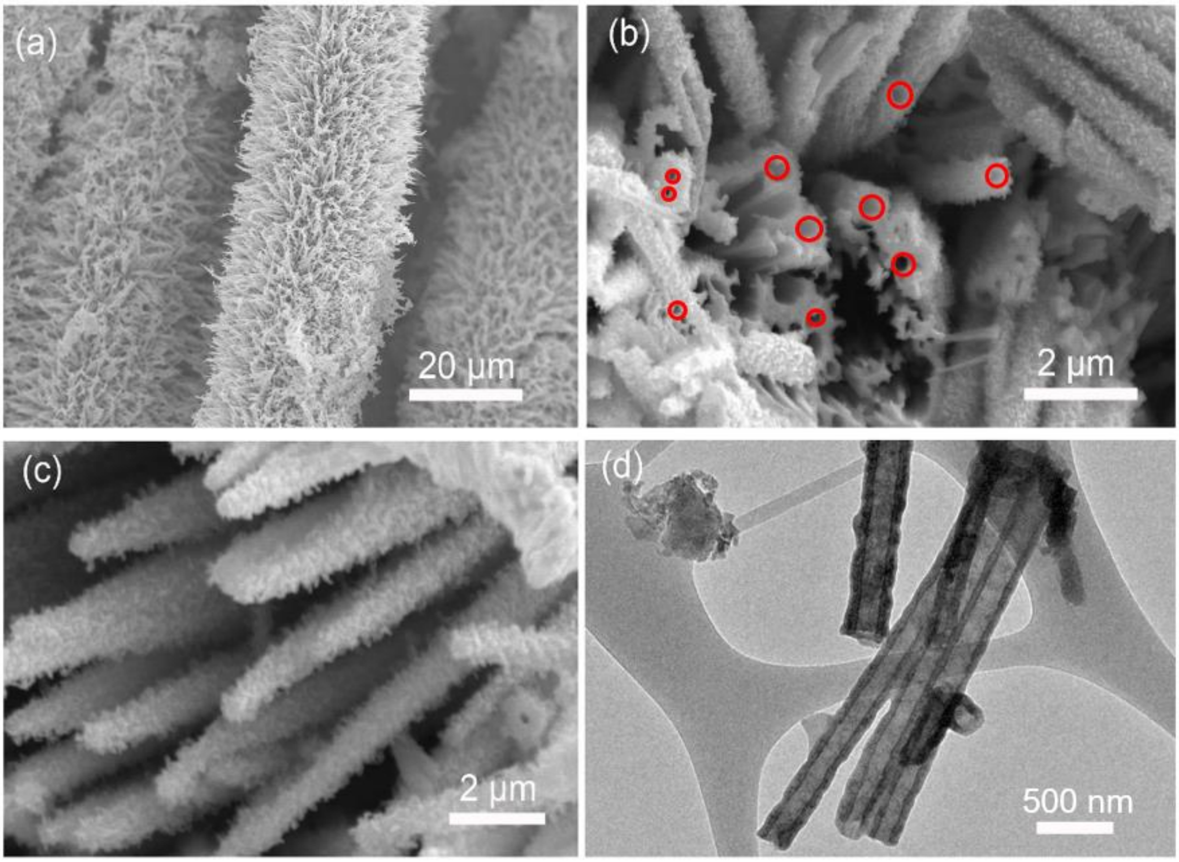

Figure S4. SEM and TEM of V-$CoSe_2$. (a) Low- resolution SEM image. (b) and (c) High-resolution SEM images. (d) TEM image. After selenization process, hollow nanotube arrays are observed in both B-$CoSe_2$ and V-$CoSe_2$ (Fig. S3b and d, Fig. S4b and d) due to the diffusion process of Co ions during the selenization driven by Kirkendall effect. And the diameter of B-$CoSe_2$ and V-$CoSe_2$ nanotubes are demonstrated to be 500 nm and 300 nm, respectively (Fig. S3b and Fig. S4b).

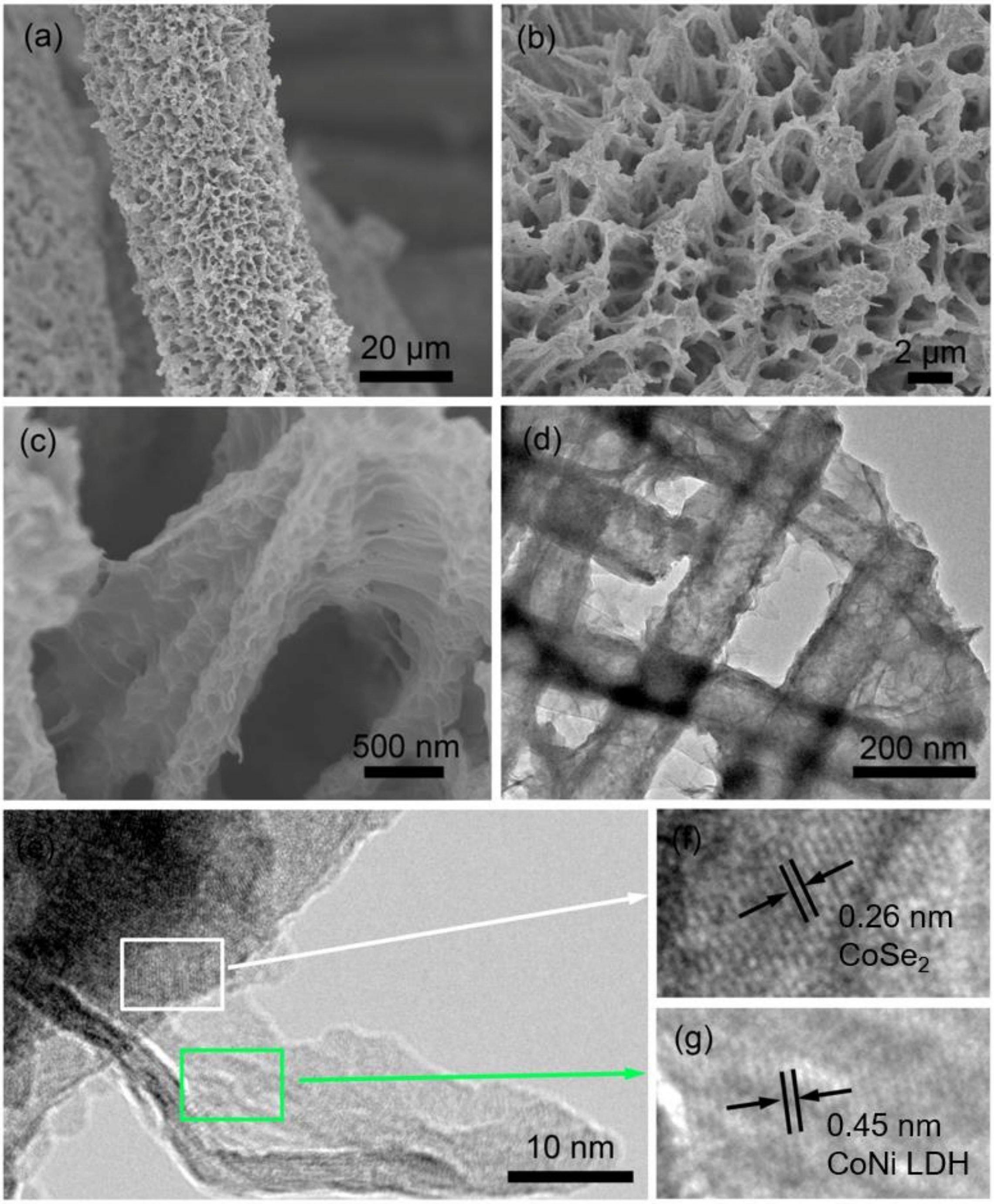

Figure S5. (a) and (b) SEM images of the as-synthesized V-$CoSe_2$@CoNi LDH HNA; (c) High-resolution SEM images of the V-$CoSe_2$@CoNi LDH HNA; and (d) TEM images of V-$CoSe_2$@CoNi LDH HNA; (e-g) HRTEM image of V-$CoSe_2$@CoNi LDH HNA highlighting the interface between nanotubes and nanosheets, with the lattice spacings of 0.26 and 0.45 nm corresponding to the (210) and (006) planes of $CoSe_2$ and CoNi LDH, respectively.

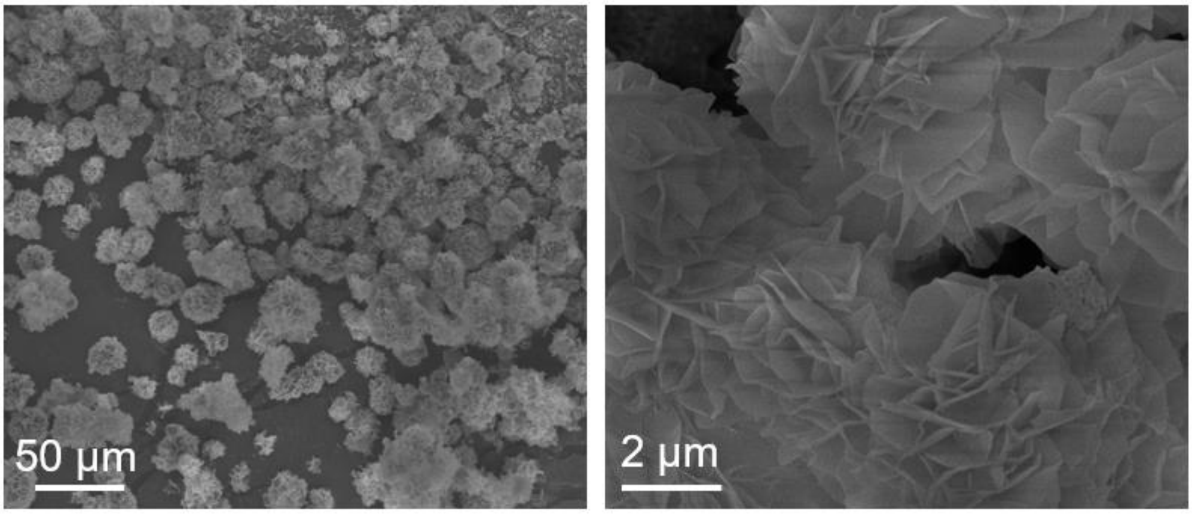

Figure S6. (a) and (b) SEM images of the CoNi LDH. The CoNi LDH sample was prepared as a reference. Compared with $CoSe_2$@CoNi LDH, the nanosheets in CoNi LDH architecture aggregate into small balls.

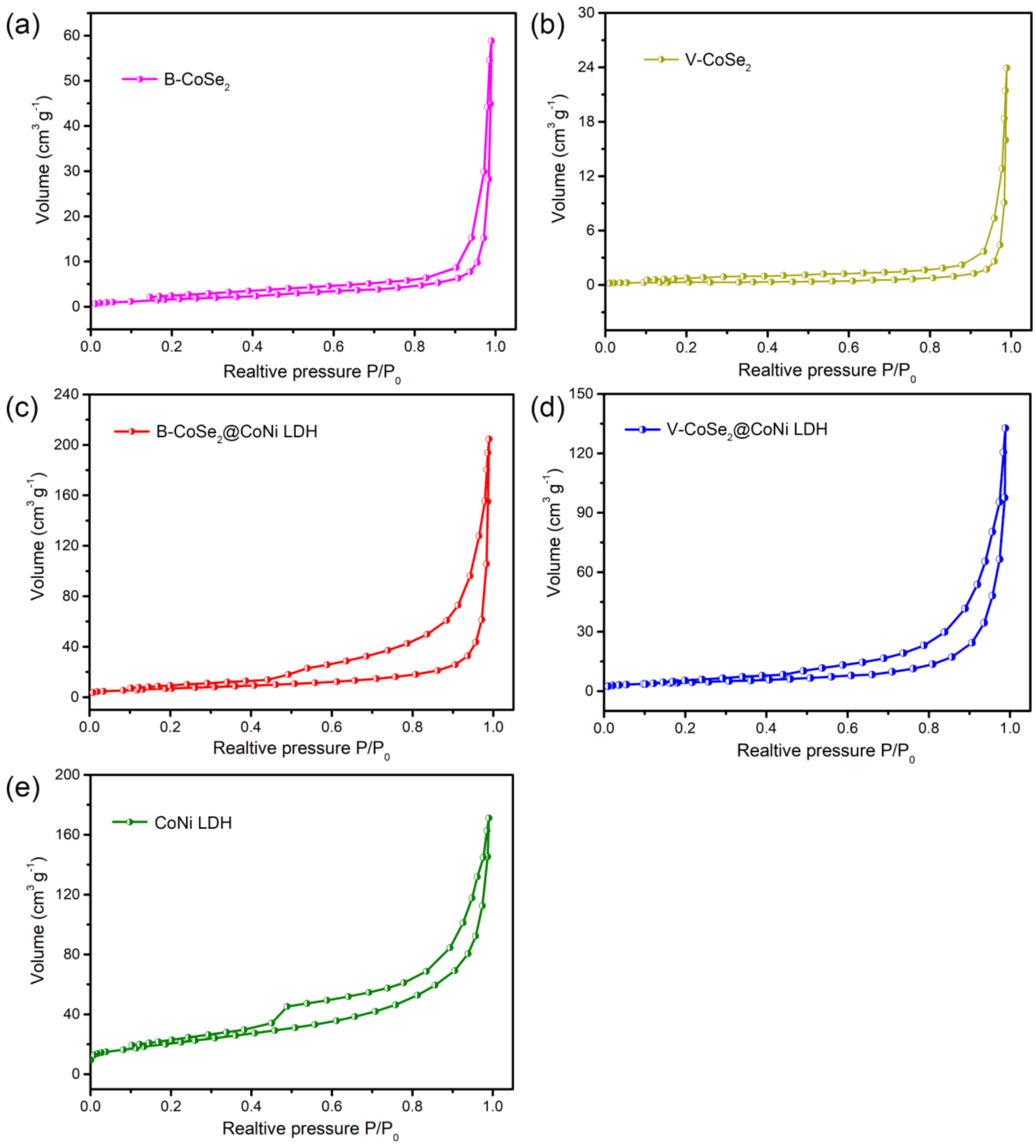

Figure S7. BET specific surface area of (a) B-$CoSe_2$, (b) V-$CoSe_2$, (c) B-$CoSe_2$@CoNi LDH HNA, (d) V-$CoSe_2$@CoNi LDH HNA and (e) CoNi LDH. The kinetics and electron transfer number (n) of several samples were further investigated, which displays the increased current density with the increase of the rotating speed due to the shortened diffusion distance at high speed.

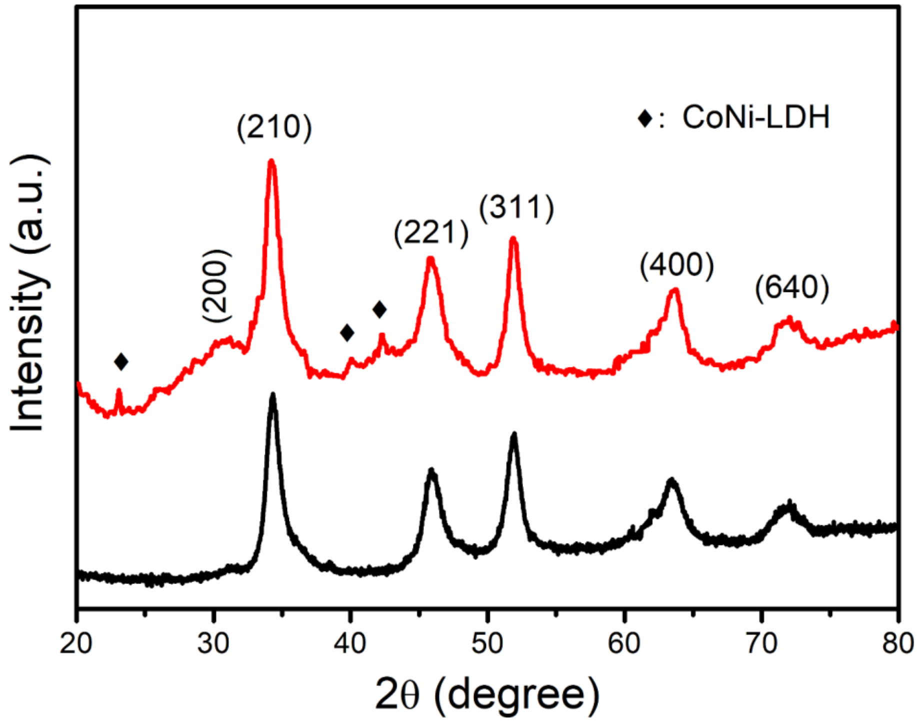

Figure S8. XRD spectra of V-$CoSe_2$ and V-$CoSe_2$@CoNi LDH HNA.

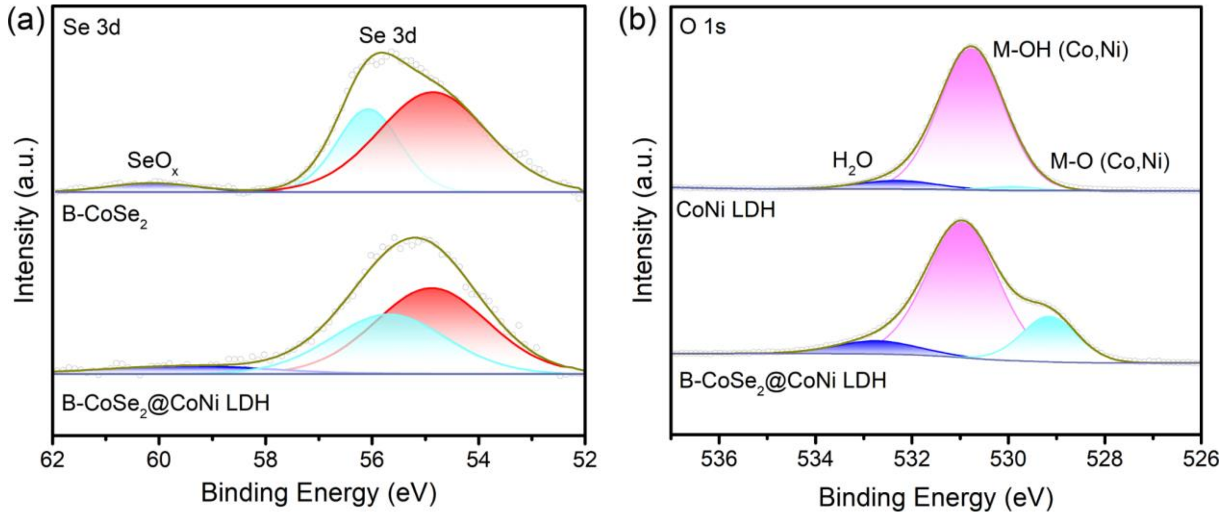

Figure S9. (a) XPS spectra of Se 3d for B-$CoSe_2$@CoNi LDH HNA and B-$CoSe_2$. (b) XPS spectra of O 1s for B-$CoSe_2$@CoNi LDH HNA and CoNi LDH.

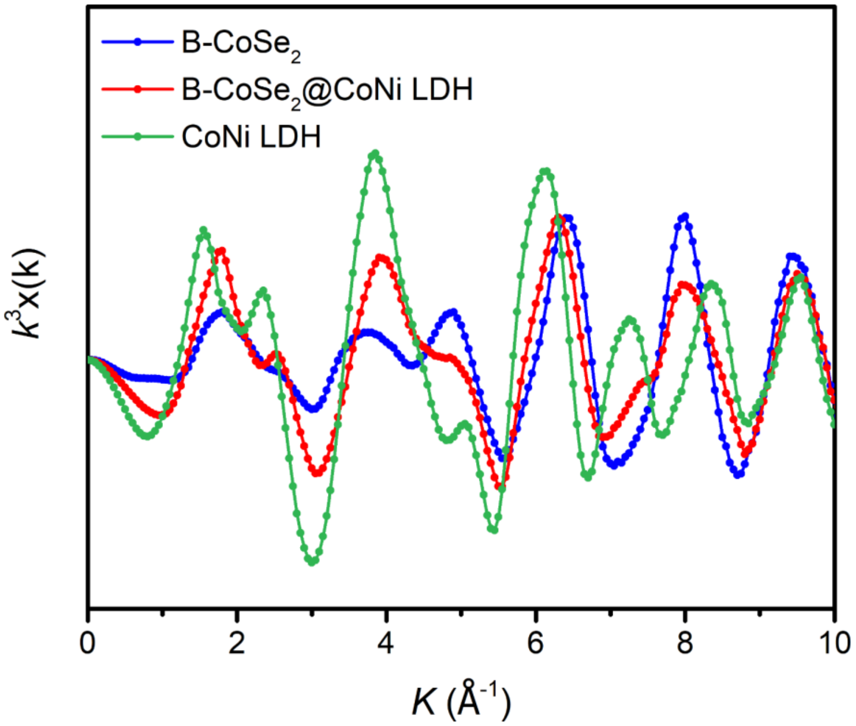

Figure S10. Co K-edge Fourier transform extended XANES oscillation function k2c(k). B-$CoSe_2$@CoNi LDH HNA shows different oscillation compared with $CoSe_2$ and CoNi LDH, indicating structure distortion after introduction of CoNi LDH nanosheets on the surface of $CoSe_2$.

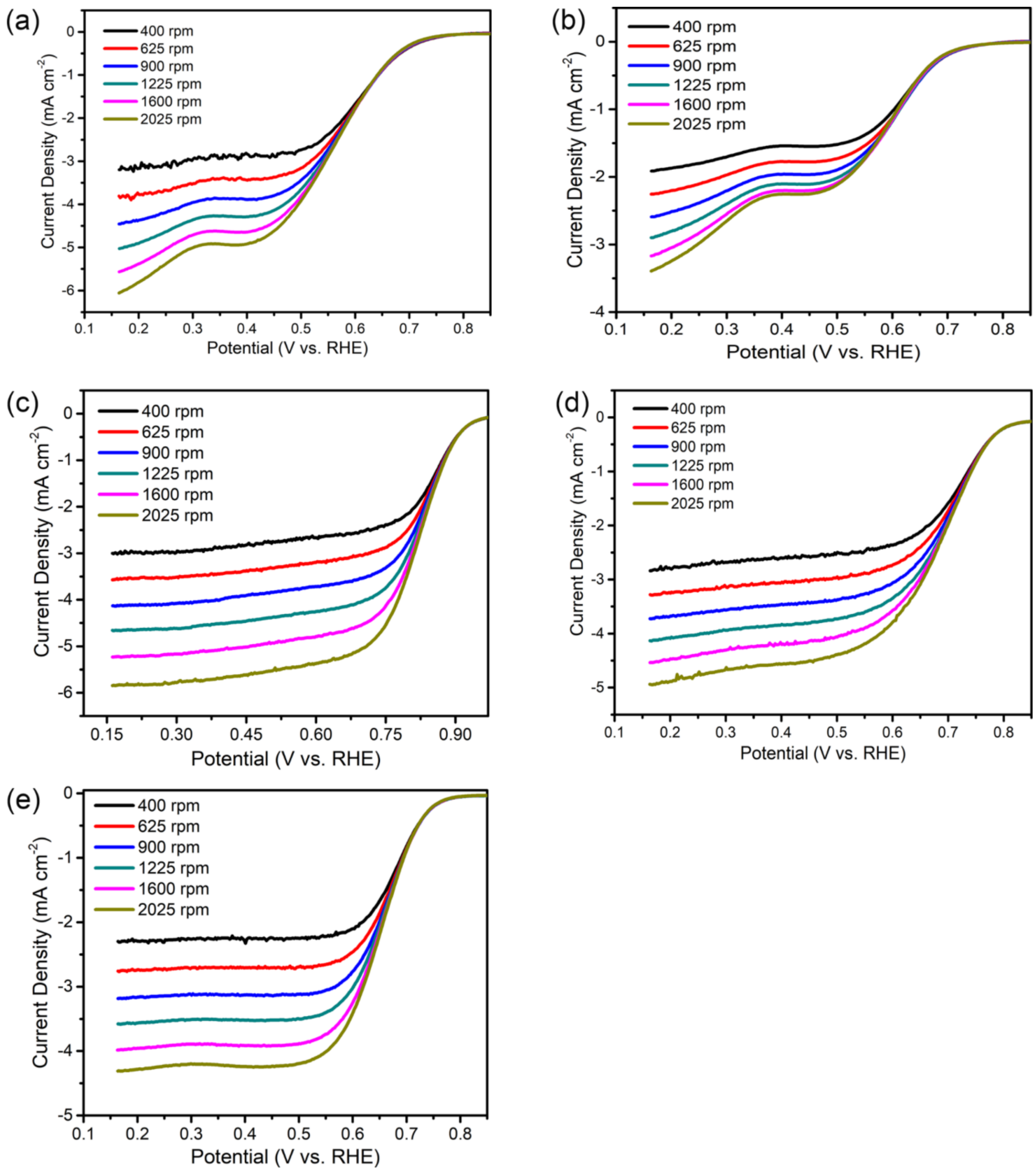

Figure S11. The ORR polarization curve of (a) B-$CoSe_2$, (b) V-$CoSe_2$, (c) B-$CoSe_2$@CoNi LDH HNA, (d) V-$CoSe_2$@CoNi LDH HNA and (e) CoNi LDH in O_2-saturated 0.1 M KOH solution at different rotation speeds (400-2025 rpm). All of the polarization curve display the increased current density with the increase of the rotating speed due to the shortened diffusion distance at high speed.

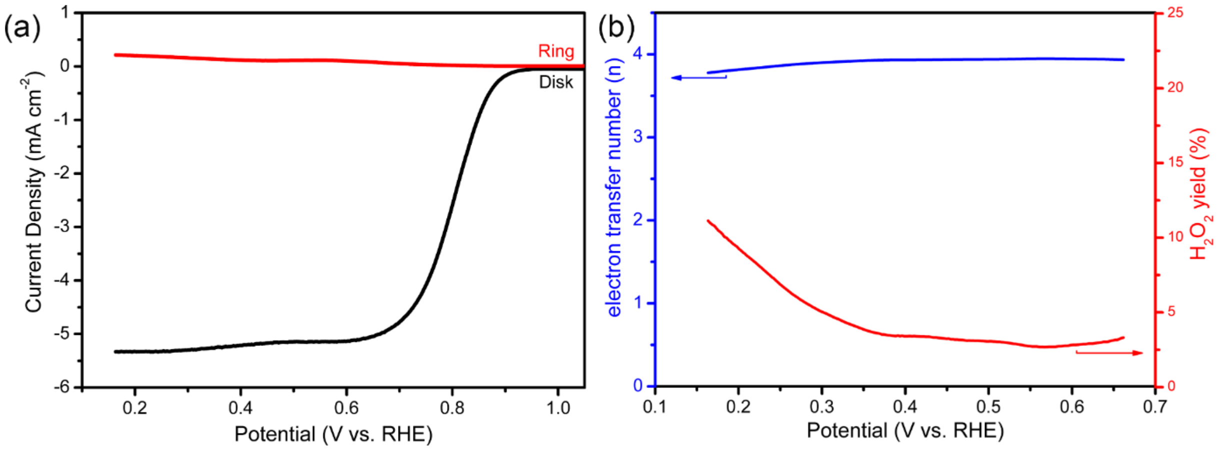

Figure S12. (a) The RRDE Polarization curve, (b) corresponding H_2O_2 yield and electron transfer number n of B-$CoSe_2$@CoNi LDH HNA during ORR. The rotating ring disk electrode (RRDE) test was also performed to examine the electrons transfer and assess the generation of peroxide (HO_2^-). The HO_2^- yield of B-$CoSe_2$@CoNi LDH HNA ranges from 2.7 % to 11 %, and the corresponding electrons transfer is in the range of 3.95 to 3.78, also indicating the four electrons transfer pathway.

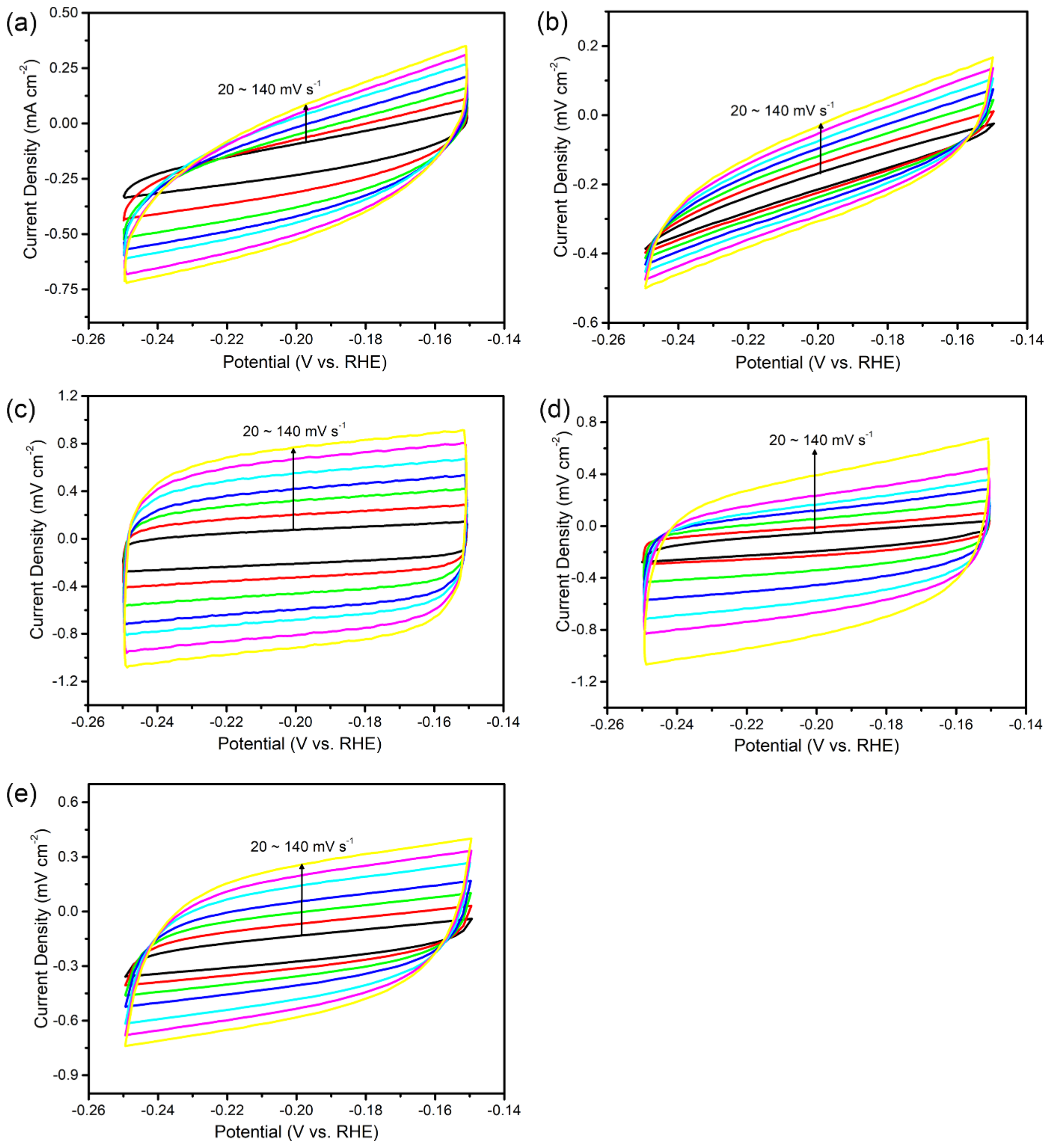

Figure S13. CV curves for (a) B-$CoSe_2$, (b) V-$CoSe_2$, (c) B-$CoSe_2$@CoNi LDH HNA, (d) V-$CoSe_2$@CoNi LDH HNA and (e) CoNi LDH samples at different scan rates from 20 to 140 mV s^{-1}, respectively.

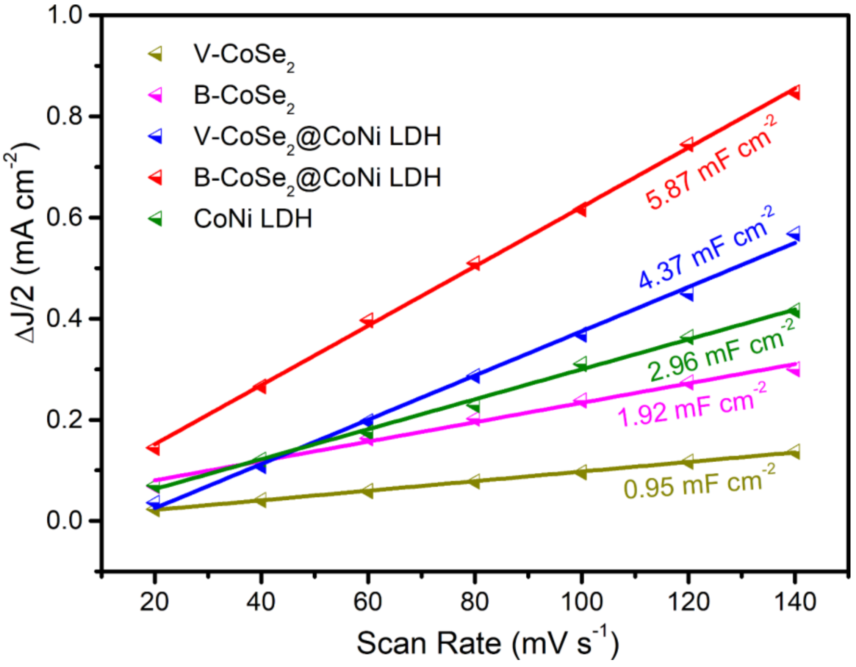

Figure S14. C_{dl} plots of B-$CoSe_2$, V-$CoSe_2$, CoNi LDH, B-$CoSe_2$@CoNi LDH HNA, V-$CoSe_2$@CoNi LDH HNA samples.

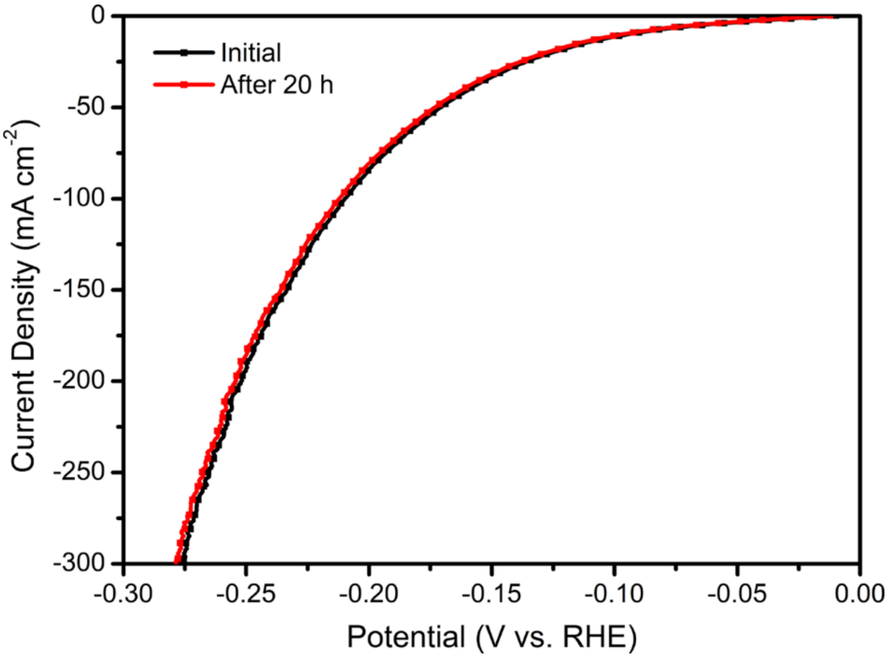

Figure S15. HER polarization curves of B-$CoSe_2$@CoNi LDH HNA catalyst before and after 20 h HER tests.

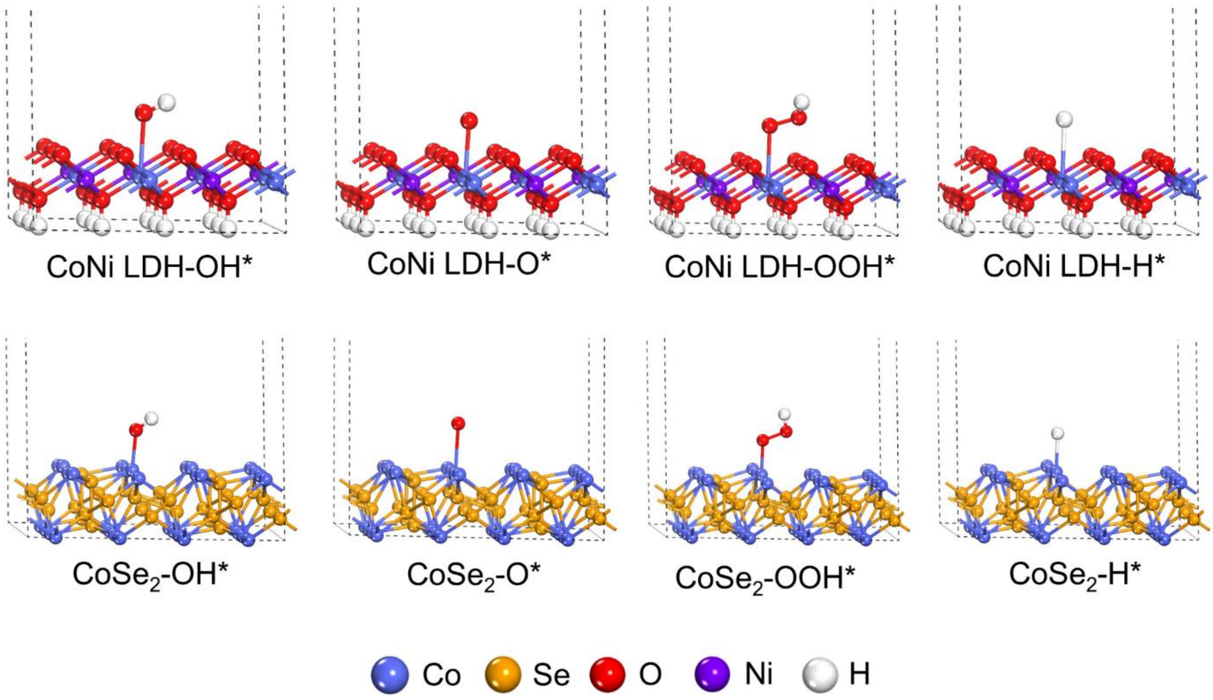

Figure S16. The DFT-optimized atomic configurations of oxygen intermediates (OOH*, O*, and OH*) and hydrogen intermediates (H*) adsorbed on $CoSe_2$ and CoNi LDH atomic interface models.

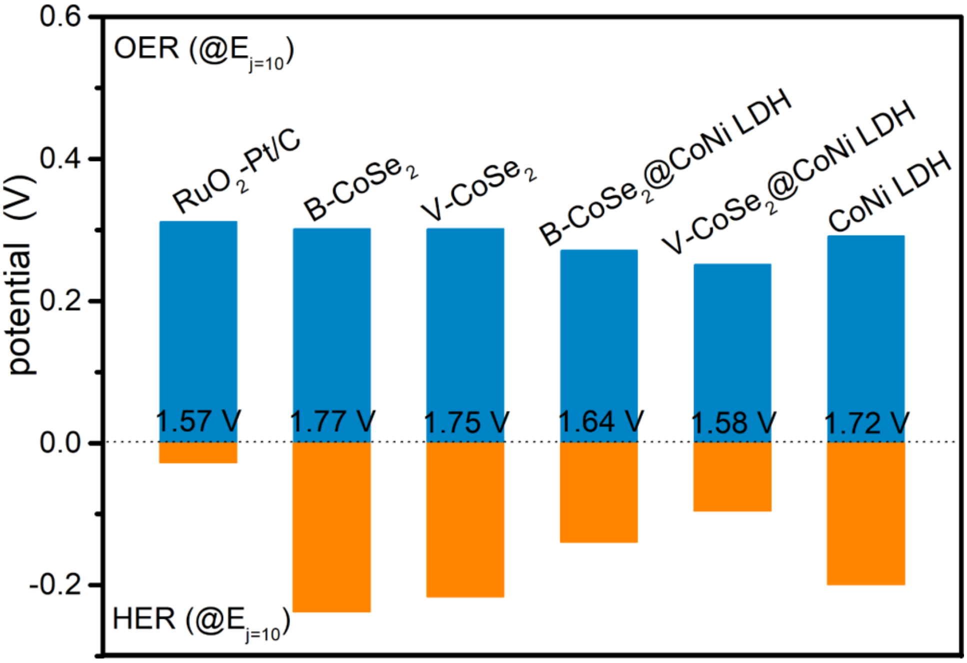

Figure S17. The overall performance comparison histogram for all catalysts by ΔE (The potential gap between HER and the OER potential at 10 mA cm^{-2}).

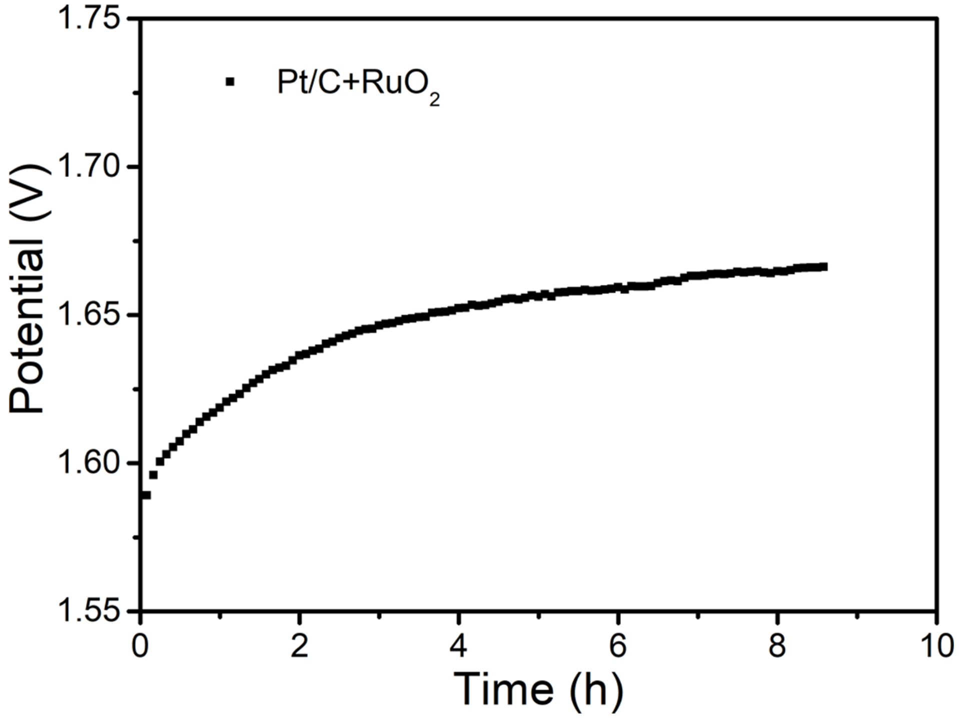

Figure S18. The stability tests of Pt/C-RuO_2 catalyst towards water splitting.

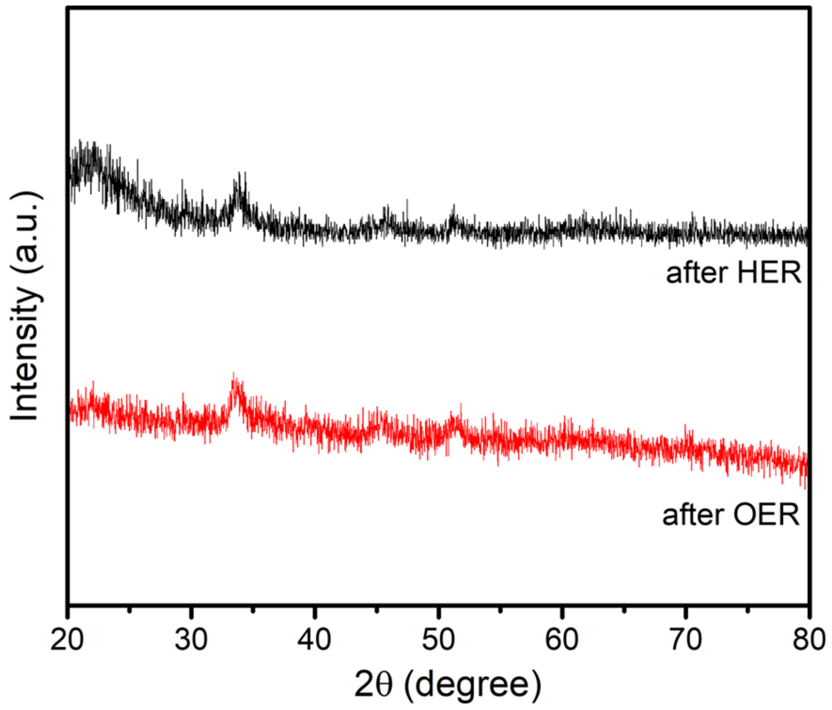

Figure S19. XRD patterns after water splitting processes for B-$CoSe_2$@CoNi LDH HNA.

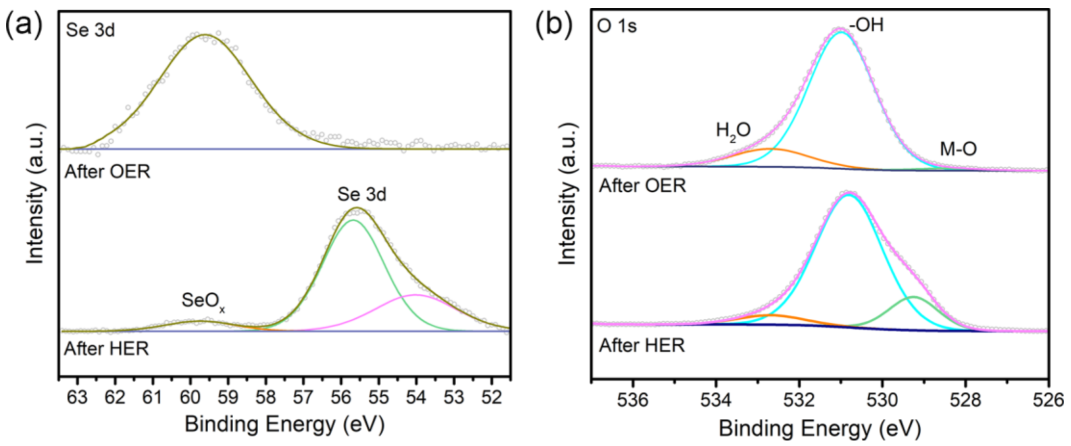

Figure S20. XPS spectra of (a) Se 3d and (b) O 1s after water splitting processes for B-$CoSe_2$@CoNi LDH HNA.

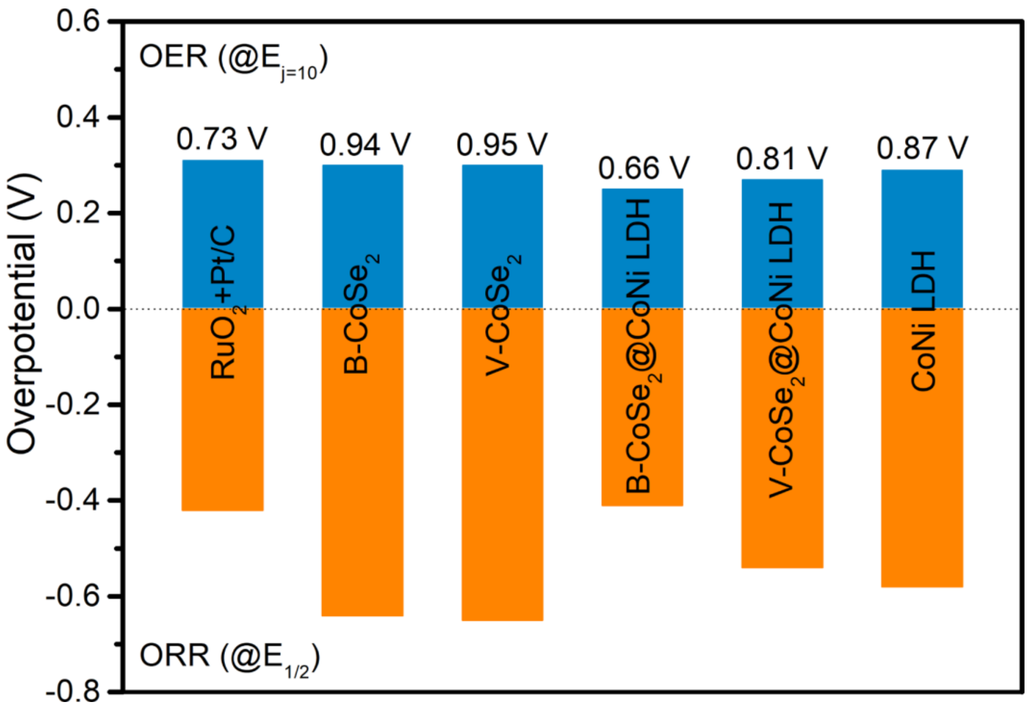

Figure S21. The overall performance comparison histogram for all catalysts by ΔE (The potential gap between ORR half-wave potential and the OER potential at 10 mA cm^{-2}).

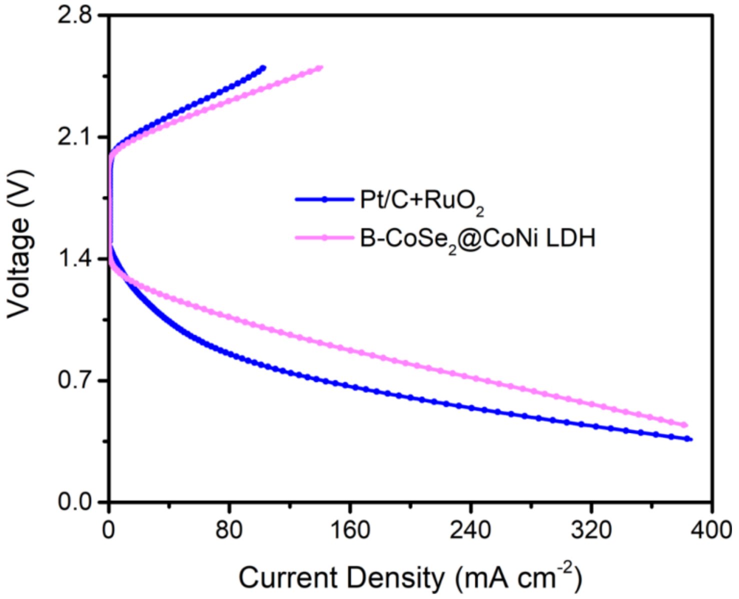

Figure S22. Charging/discharging polarization curves of B-$CoSe_2$@CoNi LDH HNA and Pt/C+RuO_2 catalysts for Zn-air batteries. A charge-discharge voltage gap of 0.8 V was obtained for B-$CoSe_2$@CoNi LDH HNA, which is lower than that of Pt/C+RuO_2 catalyst.

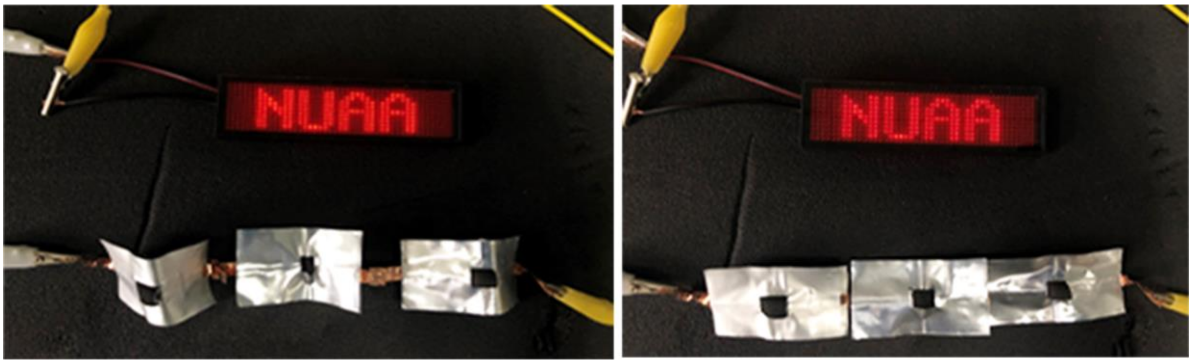

Figure S23. Photographs of a LED electronic screen driven by solid state Zn-air batteries connected in series.

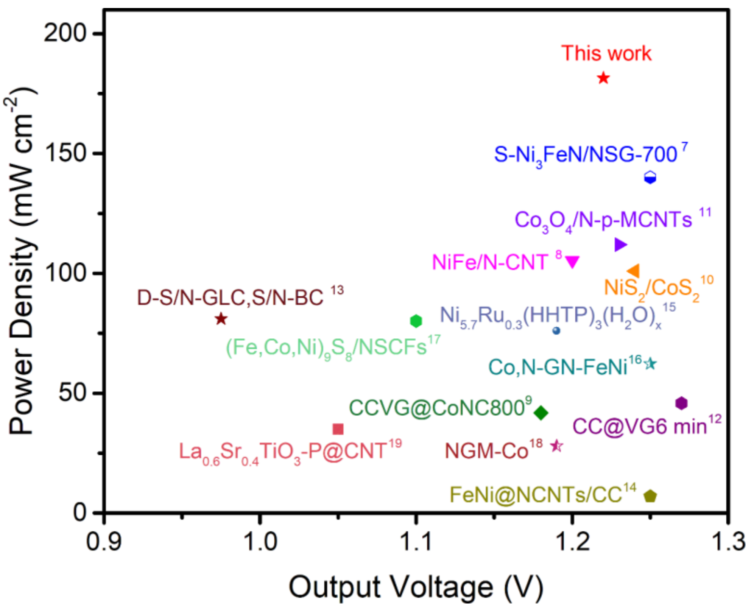

Figure S24. Ragone plots of power density and output voltage of prepared ZABs with previous ZABs.

Table S1. The comparison of OER performances of the catalysts in this work and other catalysts in the literature.

Catalysts	Substrate	*E* (mV)	Ref
**B-$CoSe_2$@CoNi LDH HNA**	**Carbon cloth**	**240 @10**	**This work**
Ni–Fe–Ce-LDH	Glassy carbon	242 @10	Energy Environ. Sci. 2020, 13, 2949-2956.
FeOOH(Se)/IF	Fe foam	287 @10	J. Am. Chem. Soc. 2019, 141, 7005-7013.
LSC&Mo_2	Ni mesh	370 @10	Nat. Commun. 2019, 10, 1723.
$CoSe_{1.26}P_{1.42}$	Glassy carbon	255 @10	ACS Energy Lett. 2019, 4, 987-994.
TiO_2@Co_9S_8	Glassy carbon	240 @10	Adv. Sci. 2018, 5, 1700772.
Fe^{2+} -NiFe LDH colloid	Carbon paper	249 @50	Angew. Chem. 2018, 130: 9536–40
NiFe LDH @Ni_3S_2/NF	Nickel foam	271 @20	Nanotechnology 2019, 30, 484001
Ultrafine monolayer NiFe LDH	Graphite paper	254 @10	Adv. Energy Mater. 2018, 8: 1703585.
NiFe LDH nanosheets	Glassy carbon	300 @10	Nat. Commun. 2014, 5: 1-9
Ni-Fe LDH hollow prisms	Glassy carbon	280 @10	Angew. Chem. Int. Ed. 2018, 57: 172.
NiFe LDH@NiCoP/NF N,S-rGO/W_2/NiFe-LDH	Nickel foam	220 @10	Adv. Funct. Mater. 2018, 28, 1706847
NiFe-LDH/Co,N-CNF	Glassy carbon	312 @10	Adv. Energy Mater. 2017, 7: 1700467.

E denote the overpotential at a current density of 10, 20, 50 mA cm^{-2}.

Table S2. The comparison of HER performances of the catalysts in this work and other catalysts in the literature.

Catalysts	Substrate	Overpotential	Ref
**B-$CoSe_2$@CoNi LDH HNA**	**Carbon cloth**	**100 mV@10**	**This work**
CoMoNiS-NF	Ni foam	166 mV@10	J. Am. Chem. Soc.2019, 141, 10417-10430.
CoFe@ NiFe LDH	Nickel foam	240 mV@10	Appl. Catal. B-Enviro.2019,253,131.
Ni_2@NC	Glassy carbon	162 mV@10	Appl. Catal. B: Environ. 2020, 272, 118976.
N-NiCoP/NF	Ni foam	78 mV@10	Appl. Catal. B: Environ. 2019, 254, 414-423.
Ti_3C_2Tx/Ni_3S_2/NF	Ni foam	72 mV@10	ACS Appl. Energy Mater. 2019, 2, 6931-6938.
NiFe LDH@NiCoP/NF	Nickel foam	120 mV@10	Adv. Funct. Mater. 2018, 28 1706847
$Ni_{0.75}Fe_{0.125}V_{0.125}$-LDHs/NF	Nickel foam	125 mV@10	Small 2018, 14, 1703257
$NiCo_2S_4$@NiFe LDH/NF	Nickel foam	200 mV@10	ACS Appl Mater Interfaces. 2017, 9, 15364
CoMoV LDH/NF	Nickel foam	270 mV@10	Chem. Commun. 2019, 55, 3521-3524
Ni_5P_4/NiP_2/NiFe LDH	Nickel foam	124 mV@10	J. Mater. Chem. A, 2018, 6,13619.
NiFeV LDH	Nickel foam	125 mV@10	Small 2018, 14, 1703257-1703266.
Co0.85Se/NiFe-LDH	Graphite foil	260 mV@10	Energy Environ. Sci. 2016, 9, 478.

Table S3. The comparison of water splitting performances of the catalysts in this work and other catalysts in the literature.

Catalysts	Substrate	potential	Ref
**B-$CoSe_2$@CoNi LDH HNA**	**Carbon cloth**	**1.58 V@10**	**This work**
NF/$Co_{5.0}Mo_1P$/NiFe-LDH	Ni foam	1.68 V@50	ACS Appl. Energy Mater. 2020, 3, 8, 8075–8085.
FeCo/Co_2P	glass carbon	1.68 V@10	Adv. Energy Mater. 2020, 1903854.
MoS_2-NiS_2/NGF/NF	Ni foam	1.64 V@10	Appl. Catal. B: Environ. 2019, 254, 15-25.
NiFeV LDH	Nickel foam	1.59 V@10	Small. 2018, 14, 1703257.
Co $(S_xSe_{1-x})_2$	Carbon fibers	1.63 V@10	Adv. Funct. Mater., 2017, 27, 1701008
$NiCo_2S_4$	Nickel foam	1.63 V@10	Adv. Funct. Mater., 2016, 26, 4661.
Cu@CoFe-LDH/Cu	Cu foam	1.68 V@10	Nano Energy, 2017, 41, 327–336.
NiFe-LDH‖Cu_3P	Cu mesh	1.72 V@10	ACS Sustainable Chem. Eng. 2018, 6, 380−388.
$NiCo_2S_4$@NiFe LDH	Glassy carbon	1.6 V@10	ACS Appl. Mater. Interfaces 2017, 9, 15364–15372.
Ni1Fe10-LDH@Ni_3S_2/NF	Ni foam	1.65 V@10	Electrochimica Acta 2019, 318, 42-50.
Co_9S_8@NiCo LDH/NF	Ni foam	1.63 V@10	Science Bulletin 2019, 64, 158–165.
$CoCO_3$@NiFe LDH	Ni foam	1.67 V@10	Materials Letters 2020, 277, 128285.
EG/$Co_{0.85}$Se/NiFe-LDH	Graphite foil	1.67 V@10	Energy Environ. Sci. 2016, 9, 478.

Table S4. Summary of the properties of transition metal compounds-based Zn-air batteries.

Air electrode	Open circuit potential (V)	Power density (mW cm^{-2})	Cycle stability (h)	Ref
**B-$CoSe_2$@CoNi LDH HNA**	**1.42**	**181.5**	**70**	**This work**
Fe-Co_4N@N-C	1.34	72	--	Appl. Catal. B-Environ. 2019, 256, 117893.
N-$NiS_{1.03}$ HS	1.41	93.9	60	Energy Storage Mater. 2020, 25, 202-209.
CoS_x/Co-NC-800	1.34	--	17	Adv. Funct. Mater. 2019, 29, 1904481
$NiCo_2S_4$/N-CNT	1.4	41.5	25	Energy Storage Materials, 2019, 16, 243.
NiO/CoN PINWs	1.46	79.6	12	ACS Nano 2017, 11, 2275.
$CuCo_2S_4$	1.2	--	22	Nanoscale, 2018, 10, 6581.
N-Co_3O_4	1.1	32	28	Adv. Mater. 2017, 29, 1602868
S-Ni_3FeN/NSG-700	1.38	140.1	35	Appl Catal B: Environ 2020, 274, 119086
Pt/NBF-ReS_2/Mo_2CTx	1.31	121.6	36	Energy storage Materials 2021, 42, 418.
IOSHs-NSC-Co9S8	1.408	--	35	Appl Catal B: Environ 2020, 260, 118209.

References

[1] H. Zhang, T. Wang, A. Sumboja, W. Zang, J. Xie, D. Gao, S.J. Pennycook, Z. Liu, C. Guan, J. Wang, *Adv. Funct. Mater.* **2018**, *28*, 1804846.

[2] F. Shen, Y. Wang, G. Qian, W. Chen, W. Jiang, L. Luo, S. Yin, *Appl. Catal. B: Environ.* **2020**, *278*, 119327.

[3] G. Kresse, J. Furthmüller, *Phys. Rev. B* **1996**, *54*, 11169.

[4] J.P. Perdew, K. Burke, M. Ernzerhof, *Phys. Rev. Lett.* **1996**, *77*, 3865.

[5] G. Kresse, D. Joubert, *Phys. Rev. B* **1999**, *59*, 1758.

[6] P. E. Blöchl, *Phys. Rev. B* **1994**, *50*, 17953.

[7] C. Lai, M. Gong, Y. Zhou, J. Fang, L. Huang, Z. Deng, X. Liu, T. Zhao, R. Lin, K. Wang, K. Jiang, H. Xin, D. Wang, *Appl. Catal. B: Environ.* **2020**, *274*, 119086.

[8] H. Lei, Z. Wang, F. Yang, X. Huang, J. Liu, Y. Liang, J. Xie, M. S. Javed, X. Lu, S. Tan, W. Mai, *Nano Energy* **2020**, *68*, 104293.

[9] Y. Zhang, H. Sun, Yunfeng Qiu, Xinyang Ji, Tiange Ma, Feng Gao, Zhuo Ma, Baoxi Zhang, PingAn Hu, *Carbon* **2019**, *144*, 370-381.

[10] Y. Cao, X. Zheng, H. Zhang, J. Zhang, X. Han, C. Zhong, W. Hu, Y. Deng, *J Power Sources* **2019**, *437*, 226893.

[11] Z. Huang, X. Qin, G. Li, W. Yao, J. Liu, N. Wang, K. Ithisuphalap, G. Wu, M. Shao, Z. Shi, *ACS Appl. Energy Mater.* **2019**, *2*, 4428–4438.

[12] B. Zhang, E. Zhang, S. Wang, Y. Zhang, Z. Ma, Y. Qiu, *J Colloid Interf. Sci.* **2019**, *543*, 84–95.

[13] J. Zhang, H. Zhou, J. Zhu, P. Hu, C. Hang, J. Yang, T. Peng, S. Mu, Y. Huang, *ACS Appl. Mater. Interfaces* **2017**, *9*, 24545–24554.

[14] X. Zhao, S. C. Abbas, Y. Huang, J. Lv, M. Wu, Y. Wang, *Adv. Mater. Interfaces* **2018**, *5*, 1701448.

[15] N. Pan, H. Zhang, B. Yang, H. Qiu, L. Li, L. Song, M. Zhang, *Chem. Commun.* **2020**, *56*, 13615-13618.

[16] T. Jiang, H. Hu, F. Lei, J. Hu, M. Wu, D. Ho, *ACS Appl. Mater. Interfaces* **2020**, *12*, 38031−38044.

[17] T. Jiang, P. Dai, W. Zhang, M. Wu, *Electrochim. Acta* **2021**, *373*, 137903.

[18] C. Tang, B. Wang, H-F Wang, Q. Zhang, *Adv. Mater.* **2017**, *29*, 1703185.

[19] K. Zeng, C. Li, J. Lu, J. Sun, X. Pan, C. Jin, M. Wei, R. Yang, Energy Fuels **2021**, *35*, 12700−12705.
